# The Primary Cilia are Associated with the Axon Initial Segment in Neurons

**DOI:** 10.1002/advs.202407405

**Published:** 2025-01-13

**Authors:** Han Wang, Yu Li, Xin Li, Zehui Sun, Fengdan Yu, Abolghasem Pashang, Don Kulasiri, Hung Wing Li, Huan Chen, Hongwei Hou, Yan Zhang

**Affiliations:** ^1^ State Key Laboratory of Membrane Biology School of Life Sciences Peking University Beijing 100871 China; ^2^ Beijing Life Science Academy Beijing 102200 China; ^3^ Centre for Advanced Computational Solutions (C‐fACS) AGLS faculty Lincoln University Canterbury 7647 New Zealand; ^4^ Department of Chemistry The Chinese University of Hong Kong Hong Kong 999077 China

**Keywords:** 5‐HT6R, axon initial segment, ciliary GPCR, primary cilia, SSTR3

## Abstract

The primary cilia serve as pivotal mediators of environmental signals and play crucial roles in neuronal responses. Disruption of ciliary function has been implicated in neuronal circuit disorders and aberrant neuronal excitability. However, the precise mechanisms remain elusive. To study the link between the primary cilia and neuronal excitability, manipulation of somatostatin receptor 3 (SSTR3) is investigated, as an example of how alterations in ciliary signaling may affect neuronal activity. It is found that aberrant SSTR3 expression perturbed not only ciliary morphology but also disrupted ciliary signaling cascades. Genetic deletion of SSTR3 resulted in perturbed spatial memory and synaptic plasticity. The axon initial segment (AIS) is a specialized region in the axon where action potentials are initiated. Interestingly, loss of ciliary SSTR3 led to decrease of Akt‐dependent cyclic AMP‐response element binding protein (CREB)‐mediated transcription at the AIS, specifically downregulating AIS master organizer adaptor protein ankyrin G (AnkG) expression. In addition, alterations of other ciliary proteins serotonin 6 receptor (5‐HT6R)and intraflagellar transport protein 88 (IFT88) also induced length changes of the AIS. The findings elucidate a specific interaction between the primary cilia and AIS, providing insight into the impact of the primary cilia on neuronal excitability and circuit integrity.

## Graphical abstract

This study identifies ciliary SSTR3 not only affects primary ciliary signaling, but also plays an important role in synaptic function, learning and memory, and neuron excitability. Furthermore, this research reveals that loss of SSTR3 leads to a decrease in Akt‐dependent CREB‐mediated transcription of AnkG. This interaction provides insight into the impact of the primary cilia on neuronal excitability and circuit integrity.

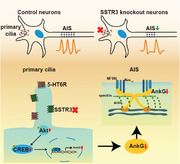

## 1 Introduction

The cilia are membrane‐bound projections from the cell surface, originating from the centrioles.^[^ ^1^ ^]^ They are categorized into two major types: motile cilia and non‐motile primary cilia.^[^ ^2^ ^]^ The primary cilia harbor numerous signal transduction components which are crucial for efficiently transmitting extracellular stimuli. Dysfunctions in the primary cilia can lead to a variety of diseases, collectively known as ciliopathies.^[^ ^3^ ^]^ Many ciliopathies are linked to dysfunctions in the nervous system, including brain dysgenesis and cognitive impairments.^[^ ^4^ ^]^ Furthermore, many neurological disorders, such as ataxia and mood disorders, are associated with abnormalities in the primary cilia.^[^ ^5^ ^]^

In the nervous system, most of the neurons possess a single primary cilium.^[^ ^6^ ^]^ Primary cilia are demonstrated to be critical for maintaining excitatory‐inhibitory balance in neuronal circuitry. One of the most common symptoms of neuronal ciliopathies is epilepsy, a neurological disorder characterized by recurrent seizures resulting from focal and widespread electrical discharges.^[^ ^7^ ^]^ Loss of ciliary GTPase in interneurons has been shown to disrupt the balance between excitatory and inhibitory activities.^[^ ^8^ ^]^ In fasting state, leptin‐induced neuronal excitability is impaired in mice with specific ciliary protein deletions.^[^ ^9^ ^]^ Moreover, recent studies have elucidated the regulatory role of ciliary ion channels in hippocampal excitability, revealing that neuronal primary cilia can function as organelle mediators of cerebral electrical signaling. The above studies indicate that the primary cilium regulates neuronal excitability and possibly could be an excitable organelle richly populated with ion channels, which contributes significantly to high‐frequency action potential (AP) firing.^[^ ^10^ ^]^

The primary cilia are enriched with numerous receptors involved in physiological functions and mediating various signaling pathways,^[^ ^11^ ^]^ which are recognized as an important nexus for G‐protein‐coupled receptor (GPCR) signaling. Numerous GPCRs and their downstream effector molecules localize to cilia on a variety of mammalian cell types.^[^ ^12^ ^]^ Notably, GPCR signaling within the cilia plays an important role in ciliopathies and neuronal excitability.^[^ ^13^ ^]^ In adult brain, the primary cilia are enriched with GPCRs sensitive to neurotransmitters, including dopamine (DA), serotonin (5‐HT), and somatostatin (SST). SST acts as a neurotransmitter in the brain, influencing cognition, motor, sensory, and autonomic function. Due to its widespread distribution, SST interacts with multiple targets through a family of five somatostatin receptor (SSTR) subtypes (SSTR1‐5), thereby exerting a broad spectrum of biological effects.^[^ ^14^ ^]^ Dysregulation of the SST‐SSTR signaling pathway has been implicated in various diseases, with studies indicating that memory loss in patients with Alzheimer's disease (AD) is associated with deficits in SST function.^[^ ^15^ ^]^

Somatostatin receptor 3 (SSTR3) stands out as a unique GPCR among the five SSTR subtypes, widely distributed throughout the brain and commonly utilized as a marker for neuronal cilia.^[^ ^16^ ^]^ SSTR3 is involved in regulating a variety of human diseases, including stomach cancer and pituitary gonadotropin adenoma.^[^ ^17^ ^]^ Additionally, SSTR3 plays a significant role in the establishment and remodeling of synaptic connectivity.^[^ ^8^, ^18^ ^]^ Disruption of SSTR3 ciliary signaling induces alterations in neuronal excitability in rat neocortical neurons. Both inhibition and activation of SSTR3‐mediated signaling can rapidly and bidirectionally regulate excitatory synapses.^[^ ^19^ ^]^ Studies involving SSTR3 knockout mice have demonstrated an increased propensity for seizures in the pentylenetetrazol model, suggesting that SSTR3 may play a role in regulating excitability of cortical neurons.^[^ ^20^ ^]^

Most of neurons receive signal inputs and generate APs at the axon initial segment (AIS), a specialized axonal compartment proximal to the soma.^[^ ^21^ ^]^ This region exhibits a high density of voltage‐gated ion channels, membrane proteins, and a unique submembrane cytoskeleton scaffold.^[^ ^22^ ^]^ The large diversity of voltage‐gated ion channel expression in the AIS is likely pivotal in dictating how various neuronal cell types transform synaptic inputs into output signals.^[^ ^23^ ^]^ Moreover, the AIS is more than a simple binary switch solely involved in AP generation. Rather, it can in addition act independently to regulate neuronal outputs in a graded analog fashion.^[^ ^24^ ^]^ Previous studies have indicated a close relationship between primary cilia and AIS. Ciliary protein localization is associated with AIS morphology.^[^ ^25^ ^]^ Ankyrin G (AnkG), a typical marker for the AIS,^[^ ^26^ ^]^ is translocated to the primary cilia of hippocampal neurons when ciliary proteins are overexpressed.^[^ ^13c^ ^]^

To gain insight into the potential association between ciliary signaling and neuronal excitability, we investigated the impact of disrupted ciliary GPCR signaling on neuronal function. Specifically, we evaluated the consequences of deleting SSTR3, which impaired primary ciliary signal transduction without physically ablating ciliary structure. This approach provided a unique model for studying the specific role of primary cilia signaling in neuronal morphology, connectivity, excitability, and synaptic integration. Our results demonstrated that the disruption of cilia signaling perturbed learning and memory, accompanied by alterations in synapse function and neuronal excitability. Notably, deficits in ciliary signaling can regulate the transcription of AIS protein, resulting in abnormal AIS structure and function. Taken together, our findings suggested a linkage between changes in primary cilia and alterations in AIS, another crucial structure for signal integration and generation, providing new insights into function of the primary cilia in the nervous system.

## 2 Results

### 2.1 Ciliary GPCR SSTR3 was Required for Maintenance of Morphology and Function of the Primary Cilia

**Figure 1:**
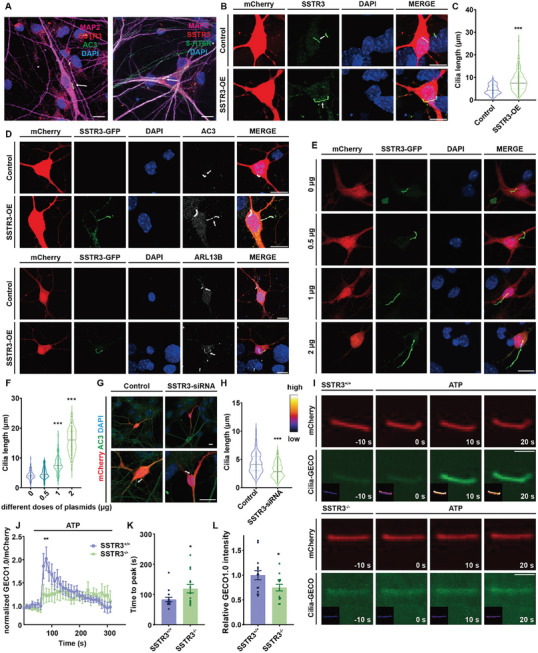
SSTR3 regulated primary cilia length and ciliary calcium signaling. A) SSTR3 localization on primary cilia in hippocampal neurons. Immunofluorescence images of primary cilia in hippocampal neurons at day 14 in vitro. Left, MAP2 (magenta) labeled neuronal microtubule‐associated protein. AC3 (green) was used to label primary cilia. SSTR3 (red) and AC3 (green) exhibited co‐localization, with DAPI (blue) labeling the nucleus. Right, MAP2 (magenta) labeled neuronal microtubule‐associated protein, 5‐HT6R (green) labeled primary cilia. SSTR3 (red) and 5‐HT6R (green) exhibited co‐localization, with DAPI (blue) labeling the nuclei. Scale bar, 10 µm. B‐D) Overexpression of SSTR3 caused an increase in primary cilia length but did not change position. B. Immunocytochemistry results for SSTR3 in the neurons of control and SSTR3 overexpression (SSTR3‐OE) at day 14. Control and SSTR3 plasmids were transfected into mouse hippocampal neurons at day 12. mCherry (red) indicated neuron morphology, SSTR3 (green) marked primary cilia, and DAPI (blue) marked the nuclei. Scale bar, 10 µm. C. Statistical data of ciliary length after transfection with control and SSTR3 plasmids. *p* < 0.001; control, n = 99; SSTR3‐OE, n = 142. Unpaired *t* test, data from 3 independent experiments. D. Immunocytochemistry results for different primary cilia markers in the neurons of control and SSTR3‐OE at day 14. Control and SSTR3‐GFP plasmids were transfected into mouse hippocampal neurons at day 12. mCherry (red) indicated neuron morphology, AC3 and ADP ribosylation factor like GTPase (ARL13B) (gray) marked primary cilia, and DAPI (blue) marked the nuclei. Scale bar, 10 µm. E‐F) Ciliary length increase was dose‐dependent on SSTR3 expression. E. Immunocytochemistry results of cilia transfected with different doses of SSTR3 plasmids (0 µg, 0.5 µg, 1 µg, and 2 µg) in mouse hippocampal neurons. mCherry (red) indicated neuron morphology, SSTR3‐GFP (green) marked primary cilia, and DAPI (blue) marked the nuclei. Scale bar, 10 µm. F. Statistical data of ciliary length after 0 µg, 0.5 µg, 1 µg, and 2 µg SSTR3‐GFP transfection. *p* < 0.001; F _(3, 481)_ = 487.1; 0 µg versus 1 µg, *p* < 0.0001. 0 µg versus 2 µg, *p* < 0.001. 0 µg, n = 96; 0.5 µg, n = 120; 1 µg, n = 124; 2 µg, n = 145. One‐way ANOVA followed by Dunnett post‐hoc test was based on data from three independent experiments. G‐H) Decrease of SSTR3 expression caused the shortening of primary cilia. G. Immunocytochemistry results for primary cilia in the neurons with control and SSTR3‐siRNA transfected. Control and SSTR3‐siRNA were transfected into mouse hippocampal neurons at day 12. mCherry (red) indicated neuron morphology, AC3 (green) marked primary cilia, and DAPI (blue) marked the nuclei. Scale bar, 10 µm. H. Statistical data of ciliary length with control and SSTR3‐siRNA treatments. *p* < 0.001; control, n = 122; SSTR3‐siRNA, n = 127. Unpaired *t* test, data from 3 independent experiments. I‐L) Defective calcium signaling was observed in SSTR3 deficient primary cilia. I. Time‐lapse imaging of hippocampal neurons from SSTR3^+/+^ and SSTR3^−/−^ mice. Neurons expressing Cilia‐GECO1.0 were treated with 20 µM ATP. Insets showed changes in fluorescence intensity in pseudocolor (white represented high and purple represented low). Scale bar, 2 µm. J. GECO1.0 fluorescence/RFP fluorescence ratio at cilia before and after administration of ATP in SSTR3^+/+^ and SSTR3^−/−^ cells. Two‐way ANOVA followed by Sidak post‐hoc test, F _(1, 4239)_ = 37.71; time 80, *p* = 0.008; SSTR3^+/+^, n = 15; SSTR3^−/−^, n = 14. The data were normalized against the average baseline values prior to ATP addition. Data shown were mean ± SEM. K. Calcium signaling in primary cilia from SSTR3^−/−^ mice exhibited delayed peak arrival time after ATP administration. *p* = 0.028; SSTR3^+/+^, n = 15; SSTR3^−/−^, n = 14. Data shown were mean ± SEM. Unpaired *t* test, data from 3 independent experiments. L. Changes in calcium indicator peak intensity at primary cilia after ATP administration in SSTR3^+/+^ and SSTR3^−/−^ mice. *p* = 0.037; SSTR3^+/+^, n = 15; SSTR3^−/−^, n = 14. The data were normalized according to the SSTR3^+/+^ group. Data shown were mean ± SEM. Unpaired *t* test, data from 3 independent experiments.

To investigate the role of cilia in neuronal activity, we first aimed to establish a system in which ciliary morphology and signaling were adjustable. It is reported that aberrant expression of ciliary proteins can alter cilium morphology which has been implicated in various human diseases.^[^ ^27^ ^]^ GPCR SSTR3 exhibited a distinct localization exclusively within the primary cilia, colocalizing with other well‐established ciliary markers such as type 3 adenylyl cyclase (AC3) and serotonin 6 receptor (5‐HT6R), as demonstrated in primarily cultured mouse hippocampal neurons (**Figure** **1**A) and in HEK293T cells (Figure S1A, Supporting Information). Our data revealed that although overexpression of SSTR3 did not alter its ciliary location (Figure 1B,D; Figure S1A, Supporting Information), it resulted in a remarkable elongation of the primary cilia (Figure 1C). Previous studies have elucidated that 4 phosphorylation sites S341, S346, S351, and T357 are critical for SSTR3 internalization and desensitization.^[^ ^28^ ^]^ Concurrently, A243, Q247, A251, Q255, F329, and K330 sites are crucial for SSTR3 ciliary localization.^[^ ^16c^ ^]^ Our results showed that disruption of SSTR3's ciliary localization through the above mutations resulted in an inability of exogenously increased SSTR3 expression to induce ciliary elongation (Figure S1B–C, Supporting Information). In order to further explore the dose dependency of SSTR3‐induced changes in cilium morphology, we transfected cultured hippocampal neurons with a series of doses of SSTR3‐GFP plasmids. As the levels of SSTR3 expression increased, there was a gradual augmentation in the length of primary cilia (Figure 1E–F).

Given that SSTR3 overexpression promoted cilia elongation, we investigated, on the other hand, whether the length of primary cilia could be shortened by down‐regulating SSTR3. Indeed, the length of the primary cilia was markedly reduced with SSTR3‐siRNA treatment (Figure 1G–H). The SSTR family has five distinct subtype members: SSTR1, SSTR2, SSTR3, SSTR4, and SSTR5. Although these five different subtypes have similar transmembrane structures, they exhibit divergent expression patterns in the central nervous system (CNS) and peripheral tissues.^[^ ^29^ ^]^ Notably, knockdown of other SSTR subtypes did not elicit significant alterations in cilium length (Figure S1F–G, Supporting Information), suggesting ciliary SSTR3 could specifically regulate cilium length.

To study if SSTR3 manipulation not only affected ciliary morphology, but also altered ciliary signaling, we used cultured hippocampal neurons from SSTR3‐knockout (SSTR3^−/−^) mice.^[^ ^20^ ^]^ Immunostaining of hippocampal sections confirmed the absence of SSTR3‐positive signal in cilia in SSTR3^−/−^ mice (Figure S1H, Supporting Information). Calcium signaling is widely used as an indicator of ciliary activity.^[^ ^30^ ^]^ We therefore examined cilia‐specific calcium dynamics in SSTR3 deficient hippocampus neurons using a genetically encoded fluorescence calcium indicator targeted selectively to the primary cilia with mCherry as a reference (Cilia‐GECO1.0).^[^ ^30c^ ^]^ ATP is known to trigger an increase in calcium level in cilia.^[^ ^30c^ ^]^ In SSTR3^+/+^ neurons, Cilia‐GECO1.0 displayed a strong activity along the primary cilia (Figure 1I). However, SSTR3^−/−^ neurons showed a remarkable reduction in calcium elevation and spent a longer time arriving at the peak compared to SSTR3^+/+^ neurons (Figure 1I–L).

The primary cilia also play critical roles in the Sonic hedgehog (SHH) signaling pathway,^[^ ^31^ ^]^ which is important during development regulating body axis formation, neuronal differentiation, bone morphogenesis,^[^ ^32^ ^]^ as well as parathyroid hormone and retinoid syntheses.^[^ ^33^ ^]^ This signaling pathway exerts its biological effects through a signaling cascade that ultimately alters the balance between active and inhibited forms of glioma‐associated oncogene (Gli) family, consisting of Gli1, Gli2, and Gli3. We did not observe significant alterations of Gli family in SSTR3^−/−^ mice hippocampal tissues and mouse embryo fibroblast (MEF) (Figure S2A–D, Supporting Information). Taken together, these data demonstrated that ciliary morphology and signaling could be perturbed by manipulating ciliary GPCR SSTR3.

### 2.2 Altered Spatial Memory and Synaptic Function were Observed in SSTR3^−/−^ Mice

**Figure 2:**
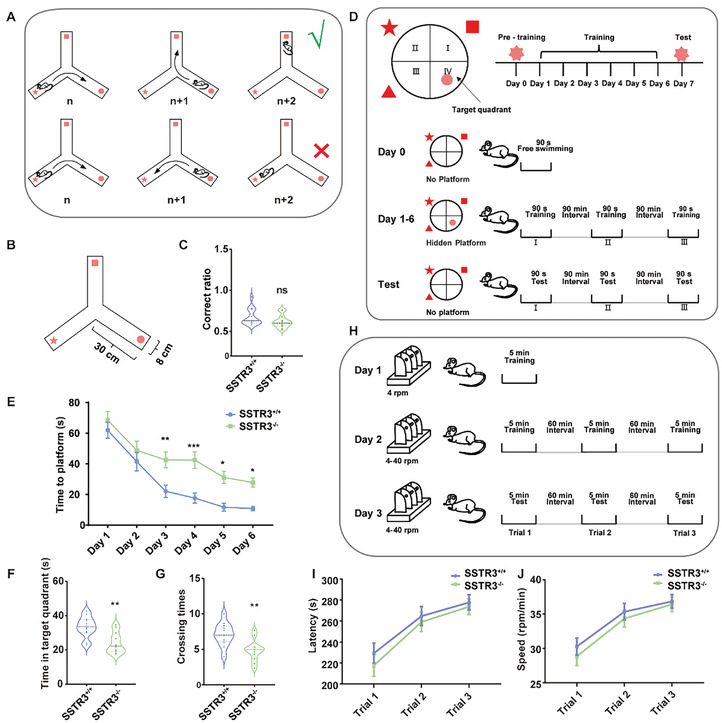
SSTR3^−/−^ mice showed spatial memory impairments. A‐B) Y‐Maze experiment judgment standard model diagram and experimental device. A. Correct exploration was indicated by the mouse choosing a different arm at the n+2 trial compared to the n trial, denoted by a green checkmark. Conversely, erroneous exploration occurred when the mouse chose the same exploration arm at the n+2 and n trials, represented by a red cross. B. The maze consisted of three exploration arms, each 30 cm in length and 8 cm in width. C) Working memory was normal in SSTR3^−/−^ mice. *p* = 0.116; SSTR3^+/+^, n = 13; SSTR3^−/−^, n = 13. Unpaired *t* test. D) Morris water maze experimental device and mode diagram. The Morris water maze experimental setup consisted of a circular pool divided into four quadrants, each marked with distinct symbols on the walls. The fourth quadrant contained a hidden platform submerged underwater. The experiment was divided into three phases: pre‐training, training, and test. During pre‐training on day 0, the platform was removed, and mice swam freely for 90 seconds to acclimate to the water environment. During the training phase from day 1 to day 6, each mouse started from different quadrants and searched for the platform in the fourth quadrant within 90 s. On the seventh day of the test, the hidden platform was removed, and mice swam freely from various quadrants for 90 seconds. The time spent by each mouse in the fourth quadrant and the number of times it crossed the original platform position were recorded to evaluate spatial memory and learning abilities. E‐G) SSTR3^−/−^ mice had impaired spatial learning and memory. E. During the training phase, SSTR3^−/−^ mice took significantly longer to reach the platform compared to SSTR3^+/+^ mice. Two‐way ANOVA followed by Sidak post‐hoc test, F _(1, 420)_ = 37.04; Day 3, *p* = 0.009; Day 4, *p* < 0.001; Day 5, *p* = 0.016; Day 6, *p* = 0.048; SSTR3^+/+^, n = 12; SSTR3^−/−^, n = 12. F. SSTR3^−/−^ mice spent less time in the target quadrant in test phase. *p* = 0.001; SSTR3^+/+^, n = 12; SSTR3^−/−^, n = 12. Unpaired *t* test. G. SSTR3^−/−^ mice had fewer crossing times. *p* = 0.002; SSTR3^+/+^, n = 12; SSTR3^−/−^, n = 12. Unpaired *t* test. H) The rotating rod experiment followed a defined flow chart. On day 1, mice underwent training with the rod rotating at a constant speed of 4 rpm for 5 min each. On day 2, the rotating rod was uniformly increased from 4 to 40 rpm, and each mouse was trained three times at 60‐minute intervals. On day 3, the rotating rod was increased from 4 to 40 rpm, and each mouse was tested three times in succession with an interval of 60 min. During these tests, the time each mouse remained on the rotating rod and the speeds at which they fell off were recorded. I‐J) The SSTR3^−/−^ mice had normal motor coordination. I. Statistical data of mouse residence time on the rotating rod. Two‐way ANOVA followed by Sidak post‐hoc test; F _(1, 78)_ = 0.964; SSTR3^+/+^, n = 12; SSTR3^−/−^, n = 12. J. Statistics of the speeds at which the mice were dropped. Two‐way ANOVA followed by Sidak post‐hoc test; F (_1, 78)_ = 0.971; SSTR3^+/+^, n = 12; SSTR3^−/−^, n = 12. The results were represented by mean ± SEM.

Human ciliopathies are associated with cognition defects.^[^ ^4c^ ^]^ Transgenic mice lacking proteins expressed in the primary cilia often exhibit defects in learning and memory.^[^ ^13^, ^34^ ^]^ Previous study showed depletion of SSTR3 results in defect in object recognition memory.^[^ ^35^ ^]^ It is widely accepted that formation of learning and memory highly depends on both neuronal excitability and synaptic connections.^[^ ^36^ ^]^ To get clues whether ciliary signaling may be associated with neuronal excitability and synaptic transmission, we employed Y‐maze test (**Figure** **2**A–B) to evaluate memory associated with working memory and the Morris water maze test (Figure 2D) to examine memory related to spatial cues in mice with deficiency in ciliary signaling. There was no statistically significant divergence in the Y‐maze test in SSTR3^−/−^ mice compared to SSTR3^+/+^ littermates (Figure 2C). However, SSTR3^−/−^ mice displayed pronounced cognitive impairment in the Morris water maze test, as evidenced by their protracted search for the submerged platform (Figure 2E). They also spent less time in the target quadrant (Figure 2F) with fewer crossing times around the platform (Figure 2G) than SSTR3^+/+^ littermates. In order to exclude the influence of motor ability, we conducted a rotarod test (Figure 2H). The results revealed that there was no significant difference in motor ability in SSTR3^−/−^ mice compared with SSTR3^+/+^ littermates (Figure 2I–J).

**Figure 3:**
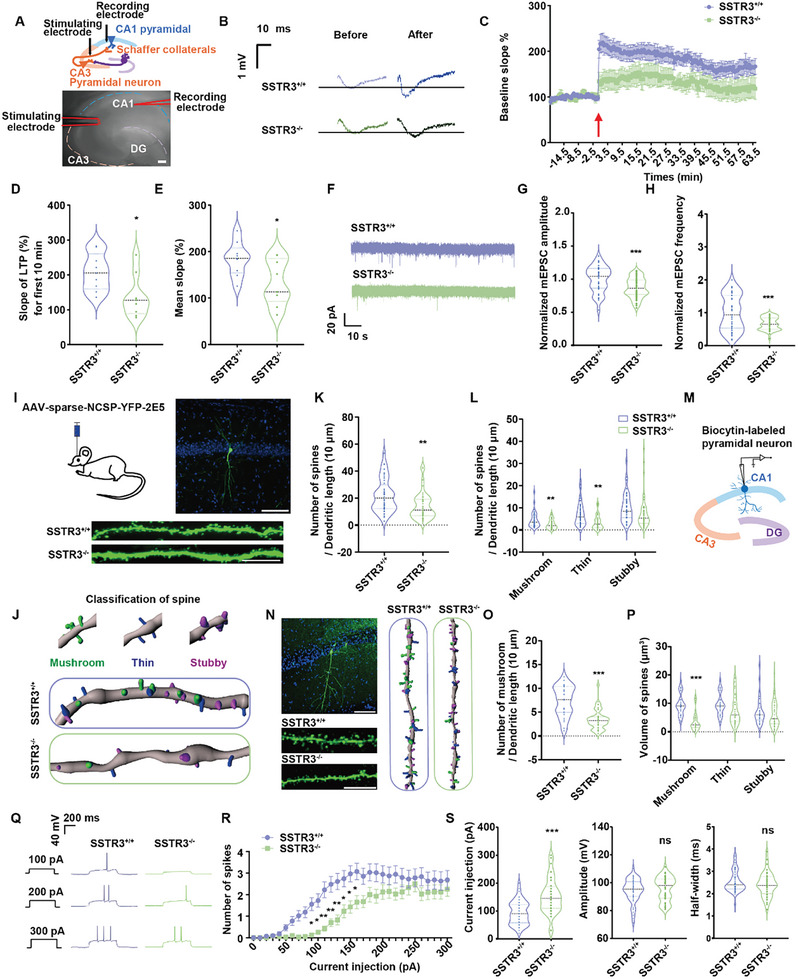
SSTR3 knockout resulted in abnormal synaptic plasticity. A) Stimulus location for LTP experiment. Above, the stimulation electrode was placed at the SC pathway of CA3 and the recording electrode was placed at the CA1 neurons. Below, typical example diagram of LTP experiment in research. Scare bar, 500 µm. B) Typical fEPSP trajectory diagram in SSTR3^+/+^ and SSTR3^−/−^ mice. C) Normalized fEPSP statistics in SSTR3^+/+^ and SSTR3^−/−^ mice. Red arrow, high frequency stimulation. Two‐way ANOVA followed by Sidak post‐hoc test; F _(1, 2720)_ = 493.2; SSTR3^+/+^, n = 9; SSTR3^−/−^, n = 9. Statistical results were normalized relative to the baseline. The result was represented by mean ± SEM. D) In the first 10 min after high‐frequency stimulation, the fEPSP slope of SSTR3^−/−^ mice was significantly reduced. *p* = 0.016; SSTR3^+/+^, n = 9; SSTR3^−/−^, n = 9. Unpaired *t* test, data from 3 independent experiments. E) Average fEPSP slope of SSTR3^−/−^ mice decreased significantly after high frequency stimulation. *p* = 0.021; SSTR3^+/+^, n = 9; SSTR3^−/−^, n = 9. Unpaired *t* test, data from 3 independent experiments. F) Typical tracks of mEPSC recorded on hippocampal neurons from SSTR3^+/+^ and SSTR3^−/−^ mice. G‐H) Amplitude and frequency of mEPSC were attenuated in SSTR3^−/−^ mice. G. Compared with SSTR3^+/+^ mice, the amplitude of mEPSC in SSTR3^−/−^ mice was significantly reduced. *p* < 0.001; SSTR3^+/+^, n = 37; SSTR3^−/−^, n = 47. Unpaired *t* test, data from 3 independent experiments. H. The frequency of mEPSC in hippocampal neurons from SSTR3^−/−^ mice was significantly reduced than that in SSTR3^+/+^ mice. *p* < 0.001; SSTR3^+/+^, n = 37; SSTR3^−/−^, n = 47. Unpaired *t* test, data from 3 independent experiments. I) Sparse‐labeling typical images. Left, illustrations of the virus injection. Right, representative images of neuronal dendrites by sparse labeling in CA1. Scare bar, 100 µm. Below, representative images of dendritic segments from two groups injected with AAV‐sparse‐NCSP‐YFP‐2E5 viruses into the hippocampus. Scare bar, 10 µm. J) Representative diagram of dendritic spines after 3D reconstruction. Above, representative images showing three types of reconstructed spines by the Imaris Spines Classifier. Spines classified as mushroom, thin, and stubby were shown in green, blue, and magenta colors, respectively. Below, representative reconstructed spine morphology of each experimental group. Each class of spine was shown with the same color in panel. K) The number of spines in SSTR3^−/−^ mice decreased significantly. *p* = 0.002; SSTR3^+/+^, n = 40; SSTR3^−/−^, n = 41. Unpaired *t* test, data from 3 independent experiments. L) Percentage of mature spines remarkably decreased in the hippocampus of SSTR3^−/−^ mice. Two‐way ANOVA followed by Sidak post‐hoc test, F _(1, 79)_ = 10.08; mushroom, *p* = 0.009; thin, *p* = 0.001; SSTR3^+/+^, n = 40; SSTR3^−/−^, n = 41. M) Diagram of biocytin‐labeled neurons by recording pipette. N) Biocytin‐labeling typical images. Left, representative images of neuronal dendrites by biocytin‐labeled in CA1. Scare bar, 100 µm. Right, representative reconstructed spine morphology of each experimental group. Each class of spine was shown with the same color in panel. Below, representative images of dendritic segments from two groups. Scare bar, 10 µm. O) The number of mushroom spines in SSTR3^−/−^ mice decreased significantly. *p* < 0.001; SSTR3^+/+^, n = 29; SSTR3^−/−^, n = 43. Unpaired *t* test, data from 3 independent experiments. P) The volume of mushroom spines remarkably decreased in the hippocampus of SSTR3^−/−^ mice. Two‐way ANOVA followed by Sidak post‐hoc test, F _(1, 70)_ = 11.91; mushroom, *p* < 0.001; SSTR3^+/+^, n = 29; SSTR3^−/−^, n = 43. Q) AP trajectories recorded on SSTR3^+/+^ and SSTR3^−/−^ mouse hippocampal neurons at day 14 in vitro. Typical trajectories with injection currents of 100 pA, 200 pA, and 300 pA were selected. R‐S) Decreased excitability of hippocampus neurons was observed in SSTR3^−/−^ mice. R. The statistical results of SSTR3^+/+^ and SSTR3^−/−^ mice showed that the number of AP burst in SSTR3^−/−^ mice was significantly lower than that in SSTR3^+/+^ mice under the same injection current. Two‐way ANOVA followed by Sidak post‐hoc test; F _(30, 2852)_ = 1.555; SSTR3^+/+^, n = 54; SSTR3^−/−^, n = 40. S. The injection current of SSTR3^−/−^ mice hippocampal neurons during the first AP burst was significantly higher than that of SSTR3^+/+^. *p* < 0.001. SSTR3^+/+^, n = 54; SSTR3^−/−^, n = 40. There was no significant difference in AP burst amplitude and AP half‐wave width between SSTR3^+/+^ and SSTR3^−/−^ hippocampal neurons. Amplitude, *p* = 0.379. Half‐wave width, *p* = 0.230; SSTR3^+/+^, n = 54; SSTR3^−/−^, n = 40. Unpaired *t* test, data from 3 independent experiments.

Memory is established in neural networks through changes in synaptic strength.^[^ ^37^ ^]^ Long‐term potentiation (LTP) is associated with synaptic plasticity and memory.^[^ ^38^ ^]^ To investigate whether the cognitive deficit in SSTR3^−/−^ mice was associated with altered hippocampal synaptic plasticity, LTP was induced at the hippocampal Schaffer collateral (SC) pathway and field excitatory postsynaptic potentials (fEPSPs) evoked at the CA1 region were recorded (**Figure** **3**A). LTP was significantly attenuated in SSTR3^−/−^ mouse hippocampal slices (Figure 3B–C), evidenced by a decrease in the magnitude of LTP compared with that in SSTR3^+/+^ littermate hippocampal slices (Figure 3D–E).

Based on our results, we further investigated whether the absence of SSTR3 led to perturbations in synaptic transmission and morphology along with impaired memory and LTP. We used whole‐cell patch clamp to record spontaneous miniature excitatory post‐synaptic currents (mEPSCs) in voltage clamp to evaluate the synaptic transmission of SSTR3^−/−^ mice. The results showed that amplitude and frequency of mEPSCs in SSTR3^−/−^ mice were significantly lower than those in control mice, revealing impaired synaptic function (Figure 3F–H). Dendritic spines are small protrusions located on neuronal dendrites and play a crucial role in synaptic function and inter‐neuronal communication. To investigate the contribution of SSTR3 to dendritic spine formation, maintenance, and structural plasticity, we examined the effects of SSTR3 knockdown on spine density and morphology in hippocampus. Dendritic spine densities were assessed using the sparse‐labeling method (Figure 3I). The fluorescent images of YFP‐labeled hippocampal neurons showed that the depletion of SSTR3 in neurons resulted in a significant loss of spines (Figure 3K). Given the diversity in dendritic spine morphology and their distinct functional properties, we classified the spines into three categories (mushroom, thin, and stubby) using the “Imaris Spines Classifier” extension (Figure 3J). The analysis revealed that SSTR3 deficiency led to marked alterations in both spine number and morphology. Specifically, SSTR3‐deficient neurons exhibited a consistent reduction in the numbers of mushroom and thin spines compared to SSTR3^+/+^ neurons (Figure 3L). Additionally, we confirmed that SSTR3 deletion affected the density of mushroom spines in CA1 region by labeling the recorded neurons with biocytin in the pipette solution (Figure 3M–P) and utilizing Golgi‐stained (Figure S3, Supporting Information). It is generally believed that mushroom‐shaped dendritic spines represent more stable and mature synapses.^[^ ^39^ ^]^ These findings suggested that SSTR3 was involved in the development, maintenance, and stability of dendritic spines, particularly those associated with mature and stable synaptic connections. Consequently, these results suggested that SSTR3 knockout impaired spatial memory and synaptic function.

Since LTP induction depends on both neuronal excitability and synaptic strength, we then examined intrinsic electrophysiological properties of neurons with ciliary perturbations. Cultured SSTR3^+/+^ and SSTR3^−/−^ mouse hippocampal neurons were injected with gradient enhanced currents from 10 to 300 pA with step enhancement amplitude of 10 pA to record membrane potential signals (Figure 3Q). Patch clamp records showed that the number of AP bursts increased with the increase of electric current, and the number of bursts in SSTR3^−/−^ mouse neurons was significantly lower than that in SSTR3^+/+^ littermate neurons (Figure 3Q–R). The results showed that the injection current of SSTR3^−/−^ mouse neurons increased significantly when they erupted APs, although amplitude of APs and half wave width were not significantly different (Figure 3S).

### 2.3 Ciliary Perturbations Led to Alterations of the AIS Structure and Function

**Figure 4:**
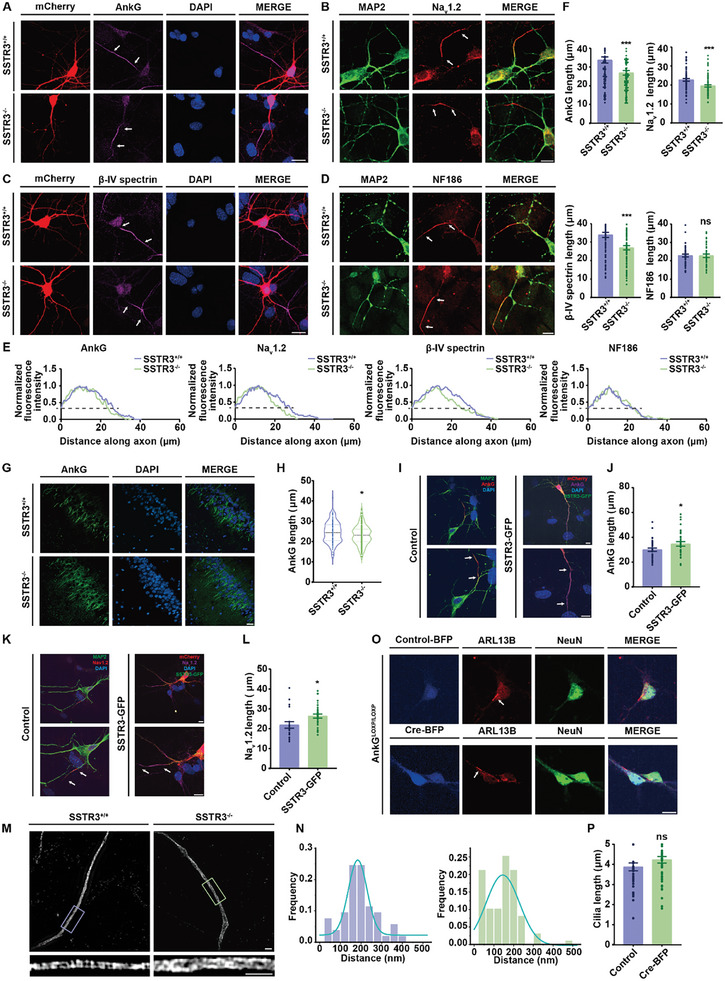
Disruption of SSTR3 reduced AIS length and impaired its structure. A‐D) The AIS length of hippocampal neurons in SSTR3^−/−^ mice was significantly reduced. AIS immunofluorescence images in SSTR3^+/+^ and SSTR3^−/−^ mouse hippocampal neurons in vitro. A.C. AnkG, and β‐IV spectrin (magenta) indicated the AIS region, mCherry (red) indicated the morphology of neurons, DAPI (blue) marked the nuclei, and white arrows indicated the AIS region. B.D. Na_v_1.2 and NF186 (red) indicated the protein positions, and MAP2 (green) indicated the morphology of dendrites and soma. Scale bar, 10 µm. E) Fluorescence intensity along the axon of AnkG, Na_v_1.2, β‐IV spectrin, and NF186 in the neurons of SSTR3^+/+^ and SSTR3^−/−^. The “start” and “end” positions of AIS were the proximal and distal axonal points, respectively, at which the profile dipped to 33% of its peak. F) Comparison of AIS‐specific protein length between SSTR3^+/+^ and SSTR3^−/−^ mice hippocampal neurons. AnkG, *p* < 0.001; SSTR3^+/+^, n = 92; SSTR3^−/−^, n = 95. Na_v_1.2, *p* < 0.001; SSTR3^+/+^, n = 70; SSTR3^−/−^, n = 73. β‐IV spectrin, *p* < 0.001; SSTR3^+/+^, n = 104; SSTR3^−/−^, n = 93. NF186, *p* = 0.943; SSTR3^+/+^, n = 55, SSTR3^−/−^, n = 40. Unpaired *t* test, data from 3 independent experiments. The results were represented by mean ± SEM. G‐H) SSTR3^−/−^ mice possessed shorter AIS than SSTR3^+/+^ mice in the hippocampus CA1 region. G. Immunostaining of CA1 region in mouse brain slices. AnkG (green) indicated the AIS, and DAPI (blue) marked the nuclei. Scale bar, 20 µm. H. Statistical data of AIS length in mouse brain slices. *p* = 0.021; SSTR3^+/+^, n = 127; SSTR3^−/−^, n = 135. Unpaired *t* test, data from 3 independent experiments. I‐J) SSTR3 expression restored the AnkG length in SSTR3^−/−^ mice. The neurons of SSTR3^−/−^ mice were transfected with control or SSTR3‐GFP plasmids at day 5. I. Immunostaining of neurons transfected with control and SSTR3 plasmids at day 7. Control, AnkG (red) indicated the AIS region, MAP2 (green) indicated the morphology of dendrites and soma, DAPI (blue) marked the nuclei, and white arrows indicated the AIS region. SSTR3‐GFP, AnkG (magenta) indicated the AIS region, mCherry (red) indicated the morphology of neurons, DAPI (blue) marked the nuclei, and white arrows indicated the AIS region. Scale bar, 10 µm. J. Statistical data of AnkG length in neurons transfected with control and SSTR3‐GFP plasmids. *p* = 0.038; control, n = 36; SSTR3‐GFP, n = 33. Unpaired *t* test, data from 3 independent experiments. The results were represented by mean ± SEM. K‐L) SSTR3 expression restored the Na_v_1.2 length in SSTR3^−/−^ mice. K. Immunostaining of neurons transfected with control and SSTR3 plasmids. Control, Na_v_1.2 (red) indicated the AIS region, MAP2 (green) indicated the morphology of dendrites and soma, DAPI (blue) marked the nuclei, and white arrows indicated the AIS region. SSTR3‐GFP, Na_v_1.2 (magenta) indicated the AIS region, mCherry (red) indicated the morphology of neurons, DAPI (blue) marked the nuclei, and white arrows indicated the AIS region. Scale bar, 10 µm. L. Statistical data of Na_v_1.2 length in neurons transfected with control and SSTR3‐GFP plasmids. *p* = 0.017; control, n = 20; SSTR3‐GFP, n = 31. Unpaired *t* test, data from 3 independent experiments. The results were represented by mean ± SEM. M‐N) AnkG showed a nonperiodic pattern in SSTR3^−/−^ neurons. M. The SIM image of AnkG in SSTR3^+/+^ and SSTR3^−/−^ mice neurons. The area in the box was shown magnified below. Scale bar, 2 µm. N. Histogram of the spacing frequency between adjacent AnkG signals. Left, SSTR3^+/+^ neurons, Gaussian fit, mean = 183 nm; single‐peak AdjR^2^ = 0.8713; histogram, n = 85. Right, SSTR3^−/−^ neurons, Gaussian fit, single‐peak AdjR^2^ = 0.57; dual‐peak AdjR^2^ = 0.75; histogram, n = 75. O‐P) The length of primary cilia was not different between control and AnkG knockout mice. O. Immunostaining of AnkG^LOXP/LOXP^ neurons transfected with control‐BFP and cre‐BFP plasmids at day 7. Control or Cre plasmid was transfected into mouse hippocampal neurons at day 3. ARL13B (red) indicated the primary cilia, and NeuN (green) indicated the neuronal nuclei. Scale bar, 10 µm. P. Statistical data of primary cilia length in neurons transfected with control‐BFP and Cre‐BFP plasmids. *p* = 0.169; control, n = 35; Cre‐BFP, n = 51. Unpaired *t* test, data from 3 independent experiments. The results were represented by mean ± SEM.

The outbreak of APs in most of the neurons occurs at the AIS.^[^ ^24^ ^]^ Given our findings indicating changes in evoked AP numbers in SSTR3^−/−^ mice, we embarked on a further exploration of the AIS alterations with ciliary perturbations. In the hippocampal neurons of SSTR3^−/−^ mice, the length of AIS was significantly shortened indicated by staining of AIS master organizer protein AnkG (**Figure** **4**A,E–F). We also observed significant shortening in other AIS‐specific proteins,^[^ ^40^ ^]^ including voltage‐gated sodium channel subtype 1.2 (Na_v_1.2) (Figure 4B,E–F) and scaffolding protein β‐IV spectrin (Figure 4C,E–F). However, extracellular matrix binding protein neurofascin‐186 (NF186) showed no difference (Figure 4D–F). We also noted that a similar phenomenon in SSTR3‐siRNA treatment, while SSTR3 overexpression led to an elongation of the AIS (Figure S4A–D, Supporting Information). The AIS length was also found to be shorter in SSTR3^−/−^ mouse brain slices (Figure 4G–H). When SSTR3 was supplemented on SSTR3^−/−^ neurons, the length of AIS was restored indicated by its associated proteins AnkG and Na_v_1.2 (Figure 4I–L). Notably, disruption of SSTR3's ciliary localization resulted in a failure of recovering AIS length (Figure S4E–F, Supporting Information), suggesting only ciliary SSTR3 specifically regulated AIS length. Knocking down other SSTR subtypes with sgRNA also did not change the length of AIS (Figure S4G–J, Supporting Information). The membrane‐associated periodic skeleton (MPS) in the axon forms a lattice structure, which has been observed by super‐resolution stochastic optical reconstruction microscopy.^[^ ^41^ ^]^ A recent study has also defined the side‐by‐side organization of actin ring structures using polarized structured illumination microscopy (SIM).^[^ ^42^ ^]^ Disturbances in MPS of AIS have been linked to various pathological conditions.^[^ ^43^ ^]^ Our SIM images of the lattice structure of the AIS indicated that AnkG showed a single‐peak distribution in control neurons. In contrast, a non‐periodic pattern was detected in SSTR3^−/−^ neurons (Figure 4M–N). However, when we observed the primary cilia of neurons with AnkG knockout (AnkG^LOXP/LOXP^), in which BFP‐tagged Cre recombinases were expressed, no significant changes were found (Figure 4O–P).

**Figure 5:**
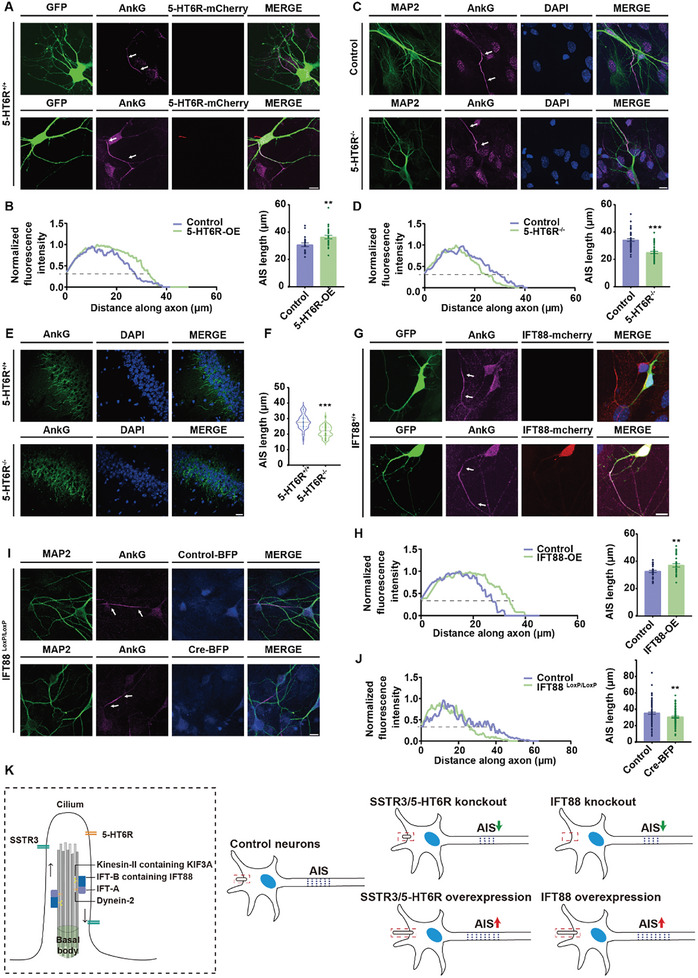
Abnormal expression of ciliary proteins affected AIS length. A‐B) 5‐HT6R overexpression led to an increase in AIS length. A. Immunostaining of neurons transfected with control and 5‐HT6R‐mcherry plasmids. AnkG (magenta) indicated the AIS region, GFP (green) indicated the morphology of neurons, and white arrows indicated the AIS region. Scale bar, 10 µm. B. Fluorescence intensity along the axon of AnkG and statistics of AIS length in neurons transfected with control and 5‐HT6R plasmids. The “start” and “end” positions of AIS were the proximal and distal axonal points, respectively, at which the profile dipped to 33% of its peak. *p* = 0.005; control, n = 21; 5‐HT6R‐OE, n = 29. Unpaired *t* test, data from 3 independent experiments. The results were represented by mean ± SEM. C‐D) The AIS length of hippocampal neurons in 5‐HT6R^−/−^ mice was significantly reduced. C.AIS immunofluorescence images in control and 5‐HT6R^−/−^ mouse hippocampal neurons in vitro. AnkG (magenta) indicated the AIS region, MAP2 (green) indicated the morphology of dendrites and soma, DAPI (blue) marked the nuclei, and white arrows indicated the AIS region. Scale bar, 10 µm. D. Fluorescence intensity along the axon of AnkG and statistics of AIS length in control and 5‐HT6R^−/−^ neurons. The “start” and “end” positions of AIS were the proximal and distal axonal points, respectively, at which the profile dipped to 33% of its peak. *p* < 0.001; control, n = 43; 5‐HT6R^−/−^, n = 45. Unpaired *t* test, data from 3 independent experiments. The results were represented by mean ± SEM. E‐F) 5‐HT6R^−/−^ mice possessed shorter AIS than control mice in the hippocampus CA1 region. E. Immunostaining of CA1 region in mouse brain slices. AnkG (green) indicated the AIS region, and DAPI (blue) marked the nuclei. Scale bar, 20 µm. F. Statistical data of AIS length in mouse brain slices. *p* < 0.001; 5‐HT6R^+/+^, n = 44; 5‐HT6R^−/−^, n = 56. Unpaired *t* test, data from 3 independent experiments. G‐H) IFT88 overexpression increased AIS length. G. Immunostaining of neurons transfected with control and IFT88 plasmids. AnkG (magenta) indicated the AIS region, GFP (green) indicated the morphology of neurons, and white arrows indicated the AIS region. Scale bar, 10 µm. H. Fluorescence intensity along the axon of AnkG and statistics of AIS length in neurons transfected with control and IFT88 plasmids. The “start” and “end” positions of AIS were the proximal and distal axonal points, respectively, at which the profile dipped to 33% of its peak. *p* = 0.005; control, n = 33; IFT88‐OE, n = 31. Unpaired *t* test, data from 3 independent experiments. The results were represented by mean ± SEM. I‐J) IFT88 knockout neurons showed shorter AIS compared to control neurons. I. Immunostaining of IFT88^LoxP/LoxP^ neurons transfected with control‐BFP and Cre‐BFP plasmids. AnkG (magenta) indicated the AIS region, MAP2 (green) indicated the morphology of dendrites and soma, and white arrows indicated the AIS region. Scale bar, 10 µm. J. Fluorescence intensity along the axon of AnkG and statistics of AIS length in neurons transfected with control and Cre plasmids. The “start” and “end” positions of AIS were the proximal and distal axonal points, respectively, at which the profile dipped to 33% of its peak. *p* = 0.007; control, n = 96; Cre‐BFP, n = 85. Unpaired *t* test, data from 3 independent experiments. The results were represented by mean ± SEM. K) The summary diagram showed that abnormal expression of ciliary proteins affected the length of AIS.

To further study the interaction between the primary cilia and AIS, we investigated the effects of manipulating other ciliary proteins on the AIS. Another ciliary GPCR, 5‐HT6R acts through an independent G protein and plays distinct physiological roles with SSTR3.^[^ ^44^ ^]^ Overexpression of 5‐HT6R increased AIS length (**Figure** **5**A–B), whereas depleting 5‐HT6R (5‐HT6R^−/−^ mice) decreased AIS length in vitro (Figure 5C–D) and in vivo (Figure 5E–F). Intraflagellar transport (IFT) is required for trafficking of proteins involved in assembly and maintenance of the primary cilium and transporting proteins that transduce cilium‐mediated signaling.^[^ ^45^ ^]^ To further study whether the interaction between the primary cilia and the AIS was associated with ciliary signal, we utilized a mouse line with a conditional knockout of IFT88, a protein essential for cilia formation and maintenance.^[^ ^3^, ^13^, ^46^ ^]^ We also observed a shortening of AIS length with deleting IFT88 using BFP‐tagged Cre recombinases, and overexpression of IFT88 caused AIS longer (Figure 5G–J). These results suggested that alterations in the cilia affected the AIS structure (Figure 5K).

**Figure 6:**
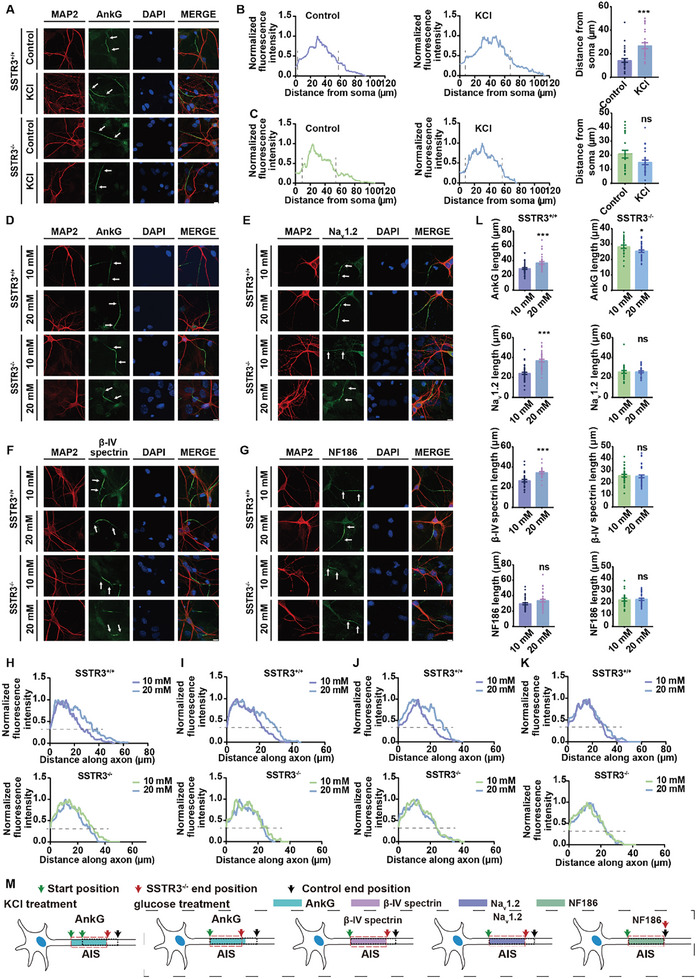
Disruption of SSTR3 impaired AIS plasticity. A‐C) AIS position plasticity disappeared in SSTR3^−/−^ neurons in 15 mM K^+^ condition. A. AnkG immunofluorescence images in SSTR3^+/+^ and SSTR3^−/−^ mouse hippocampal neurons at day 14. The treatment group of neurons was exposed to 15 mM extracellular potassium for a duration of 2 days. AnkG (green) indicated the AIS region, MAP2 (red) indicated the morphology of dendrites and soma, DAPI (blue) marked the nuclei, and white arrows indicated the AIS region. Scale bar: 10 µm. B. Fluorescence intensity of AnkG from soma in the neurons of SSTR3^+/+^. The “start” and “end” positions of AIS were the proximal and distal axonal points, respectively, at which the profile dipped to 33% of its peak. Statistical graph, *p* < 0.001; control, n = 32; KCl, n = 24. C. Fluorescence intensity of AnkG from soma in SSTR3^−/−^ neurons. Statistical graph, *p* = 0.056; control, n = 23; KCl, n = 30. Unpaired *t* test, data from 3 independent experiments. The results were represented by mean ± SEM. D‐G) AIS length plasticity was impaired in SSTR3^−/−^ neurons. Immunocytochemistry results for AnkG, Na_v_1.2, β‐IV spectrin, and NF186 in the neurons of SSTR3^+/+^ and SSTR3^−/−^ at day 7. The neurons in the control group were cultured in a medium containing 10 mM glucose, while the experimental group was treated with a medium containing 20 mM glucose for a duration of 10 h. AnkG, Na_v_1.2, β‐IV spectrin, and NF186 (green) indicated the AIS region, MAP2 (red) indicated the morphology of dendrites and soma, DAPI (blue) marked the nuclei, and white arrows indicated the AIS region. Scale bar, 10 µm. H‐K) Control neurons possessed longer AIS in 20 mM glucose than in 10 mM, whereas the SSTR3^−/−^ neurons did not show this difference. H. Fluorescence intensity of AnkG along axon in the neurons of SSTR3^+/+^ and SSTR3^−/−^ with treatment of glucose. I. Fluorescence intensity of Na_v_1.2 along axon in the neurons of SSTR3^+/+^ and SSTR3^−/−^ with treatment of glucose. J. Fluorescence intensity of β‐IV spectrin along axon in the neurons of SSTR3^+/+^ and SSTR3^−/−^ with treatment of glucose. K. Fluorescence intensity of NF186 along axon in the neurons of SSTR3^+/+^ and SSTR3^−/−^ with treatment of glucose. L) Statistical graphs of AIS protein length in SSTR3^+/+^ and SSTR3^−/−^ neurons with glucose treatment. AnkG, left, SSTR3^+/+^ neurons, *p* = 0.0008; 10 mM, n = 43; 20 mM, n = 33; right, SSTR3^−/−^ neurons, *p* = 0.029; 10 mM, n = 29; 20 mM, n = 35. Na_v_1.2, left, SSTR3^+/+^ neurons, *p* < 0.001; 10 mM, n = 41; 20 mM, n = 35; right, SSTR3^−/−^ neurons, *p* = 0.940; 10 mM, n = 29; 20 mM, n = 31. β‐IV spectrin, left, SSTR3^+/+^ neurons, *p* < 0.001; 10 mM, n = 32; 20 mM, n = 24; right, SSTR3^−/−^ neurons, *p* = 0.797; 10 mM, n = 36; 20 mM, n = 41. NF186, left, SSTR3^+/+^ neurons, *p* = 0.083; 10 mM, n = 35; 20 mM, n = 37; right, SSTR3^−/−^ neurons, *p* = 0.790; 10 mM, n = 24; 20 mM, n = 33. Unpaired *t* test, data from 3 independent experiments. The results were represented by mean ± SEM. M) AIS plasticity diagram in control and SSTR3^−/−^ mice. The colored rectangles represented the original position of AIS proteins, the black dotted box represented the position of AIS after plasticity occurred in the control group, and the red dotted box represented the position after plasticity occurred in SSTR3^−/−^ mice.

A change in position as well as a change in length along the AIS, so‐called AIS plasticity, was originally reported in response to alterations in neuronal activity and has been proposed to act as stabilizers of neuronal excitability.^[^ ^47^ ^]^ For changes in AIS position (here named position plasticity), chronic depolarization with high extracellular potassium moves multiple components of the AIS, up to 17 µm away from the soma of excitatory neurons. This mechanism allows neurons to regulate the position of an entire subcellular structure according to their patterns of electrical activity.^[^ ^47a^ ^]^ Here, we also found that treatment of 15 mM extracellular potassium from 12 to 14 days in vitro induced changes in the position of AIS (**Figure** **6**A–B). Strikingly, we found that AIS position plasticity was absent in SSTR3^−/−^ mice (Figure 6A–C). We have applied different concentrations of glucose to induce AIS length change (here named length plasticity) in our previous studies.^[^ ^48^ ^]^ The treatment of dissociated hippocampal neurons with 20 mM glucose for 10 h resulted in a significant increase in the length of the AIS compared to the control 10 mM group. This was observed through AnkG immunolabeling, indicating an alteration in AIS morphology in response to the glucose treatment (Figure 6D,H,L). Similar changes were observed in the other two AIS proteins, Na_v_1.2 and β‐IV spectrin, but not NF186 (Figure 6E–G). However, glucose treatment shortened AnkG length and left other AIS proteins unchanged in the neurons from SSTR3^−/−^ mice (Figure 6D–K). These results suggested that abnormalities in cilia‐related protein SSTR3 affected AIS structure and function (Figure 6M).

### 2.4 Ciliary GPCR Deficiency Led to Distinctive Alterations in Gene Expression

**Figure 7:**
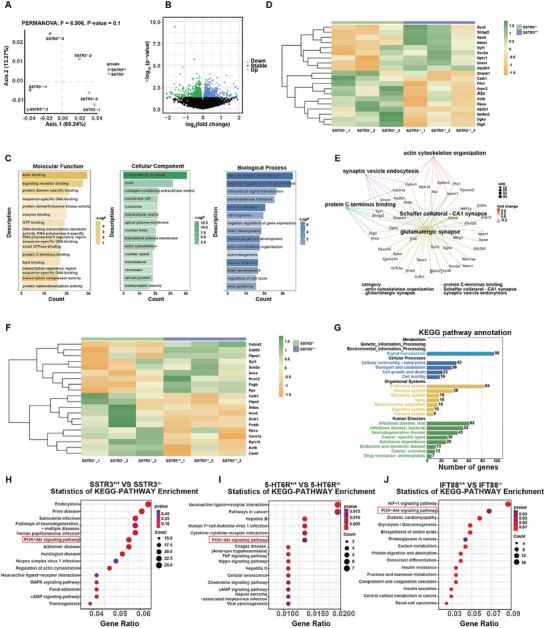
SSTR3 deficiency led to distinctive alterations in gene expression. A) PCA analysis with PERMANOVA revealed distinct clustering for SSTR3^+/+^ and SSTR3^−/−^ mice. The purple and green dots indicated the SSTR3^+/+^ group and SSTR3^−/−^ group, respectively. The major Axises were 69.24% and 13.27%, F = 6.906, *p*‐value = 0.1, n = 3. B) Volcano plot showed genes that were significantly differentially expressed in SSTR3^+/+^ and SSTR3^−/−^ mice. The x‐axis and y‐axis were represented by log_2_(fold change) and ‐log_10_(*p*‐value). The black dots indicated that these genes were not part of the screening criteria (*p*‐value ≤ 0.05). Other genes were represented as green (< 0) and blue (> 0) based on the value of log_2_ (fold change). n = 3 for each group. C) GO enrichment analysis showed the DEGs were mainly connected with glutamatergic synapse and axon. The Count (X‐axis) indicated the number of genes contained in a GO term. D) Clustering heatmap showed the top 20 DEGs about glutamatergic synapse. The color filling of rectangles was determined based on the log_2_(fold change) of genes, progressing from orange (< 0) to green (> 0). E) A gene‐concept network showed the interplay between the DEGs in glutamatergic synapse and their associated GO terms. The size of nodes indicated the number of genes contained in a GO term. The color filling of genes was determined based on the log_2_(fold change) of genes, progressing from purple (< 0) to red (> 0). Category showed the top 5 terms for GO enrichment analysis using the DEGs significantly associated with glutamatergic synapse. F) Clustering heatmap showed the top 20 DEGs about axon. The color filling of rectangles was determined based on the log_2_(fold change) of genes, progressing from orange (< 0) to green (> 0). G) Key KEGG pathways annotation of the DEGs in SSTR3^+/+^ and SSTR3^−/−^ mice. The KEGG pathways were classified into three levels according to the classification page on the KEGG pathway database. The second level pathways that were identified in the KEGG enrichment analysis were summarized (color) and viewed on the first level (black). H) KEGG pathway enrichment analysis of DEGs in SSTR3^+/+^ and SSTR3^−/−^ mice. The Count indicated the number of genes contained in a KEGG pathway. The Gene Ratio (X‐axis) indicated the number of genes contained in a KEGG pathway as a proportion of all DEGs. The red dotted box represented the intersection in H‐J. I) KEGG pathway enrichment analysis of DEGs in 5‐HT6R^+/+^ and 5‐HT6R^−/−^ mice. The Count indicated the number of genes contained in a KEGG pathway. The Gene Ratio (X‐axis) indicated the number of genes contained in a KEGG pathway as a proportion of all DEGs. The red dotted box represented the intersection in H‐J. J) KEGG pathway enrichment analysis of DEGs in IFT88^+/+^ and IFT88^−/−^ mice. The Count indicated the number of genes contained in a KEGG pathway. The Gene Ratio (X‐axis) indicated the number of genes contained in a KEGG pathway as a proportion of all DEGs. The red dotted box represented the intersection in H‐J.

In order to explore potential targets of how ciliary SSTR3 may involve in cilium morphology, cognitive impairment, and AIS alterations, we performed RNA sequencing (RNA‐seq) of hippocampal tissues from 6‐month‐old adult control and SSTR3^−/−^ mice. The RNA‐seq datasets have been deposited in Gene Expression Omnibus (GEO): GSE248779. We applied Principal Component Analysis (PCA) which revealed distinct clustering for each experimental group (**Figure** **7**A). This PCA analysis underscored the marked discrepancies in gene expression profiles. Among 15837 genes investigated, we identified 985 differentially expressed genes (DEGs; *p* < 0.05) between the control and SSTR3^−/−^ mice (Figure 7B). To gain a deeper insight into the functional implications of these DEGs, we performed Gene Ontology (GO) analysis. The top 15 enriched GO terms of molecular function, cellular component, and biological process were illustrated (Figure 7C). Notably, these DEGs exhibited enrichments in nervous system development and played pivotal roles in synaptic and axonal functions. These were consistent with the results that synapses and axons were affected by the SSTR3 disturbance. We screened the top 20 DEGs associated with synapses (Figure 7D). *Syn2*, *Neto1*, and *Syt1* were among them. *Syn2* deletion produces epileptic seizures and overexcitability in neuronal networks. It is also important for neurite growth.^[^ ^49^ ^]^ *Neto1*‐null mice have depressed LTP at Schaffer collateral‐CA1 synapses and impaired spatial learning and memory.^[^ ^50^ ^]^ Patients with *Syt1*‐associated neurodevelopmental disorders demonstrate infantile hypotonia, profound intellectual disability, disordered movement, and electroencephalographic abnormalities without epilepsy or gross neuroanatomic abnormalities.^[^ ^51^ ^]^ The gene‐concept network we generated highlighted the interplay between specific genes and their associated GO terms. Notably, the network revealed the centrality of glutamatergic synapses and Schaffer collateral‐CA1 synapse‐related concepts, indicating their importance in the regulatory network (Figure 7E). In the heat map of axon‐related DEGs, several genes associated with cell excitability, including *Snca2a* and *Kcnc2*,^[^ ^52^ ^]^ were prominently displayed (Figure 7F). Collectively, our results suggested that various genes involved in synaptic plasticity, neuronal excitability, and axon function were altered in SSTR3^−/−^ mice.

The Kyoto Encyclopedia of Genes and Genomes (KEGG) pathway annotation revealed that the DEGs were enriched in signal transduction (Figure 7G), and the KEGG analysis suggested that the regulatory pathways exhibiting significant differences included endocytosis, PI3K‐Akt signaling pathway, neuroactive ligand‐receptor interaction, and cAMP signaling pathway in control and SSTR3^−/−^ mice (Figure 7H). Using online 5‐HT6R knockout RNA‐seq data (GSE137942) that we previously reported ^[^ ^13d^ ^]^ and IFT88 knockout RNA‐seq data (GSE137177), we performed KEGG analysis about 5‐HT6R and IFT88 knockout (Figure 7I–J). Among the top ten significantly different expression pathways, we found the intersection PI3K‐Akt signaling pathway.

### 2.5 SSTR3 and 5‐HT6R Ablation Suppressed CREB Activity and its Downstream Gene AnkG Transcription

**Figure 8:**
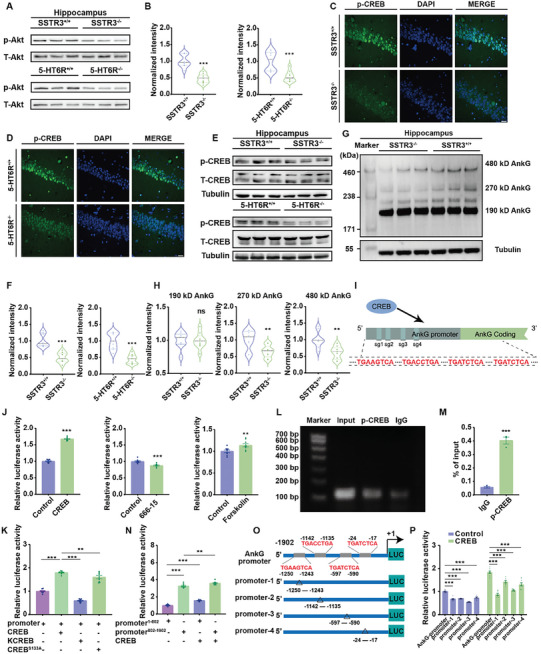
CREB bound with AnkG promoter to regulate AnkG transcription. A‐B) The expression level of p‐Akt/T‐Akt in hippocampus from SSTR3^−/−^ mice and 5‐HT6R^−/−^ mice were significantly decreased compared to control mice. A. Western blotting of p‐Akt and T‐Akt expression of hippocampus. B. Quantitative analysis of p‐Akt and T‐Akt expression ratio. SSTR3^+/+^ versus SSTR3^−/−^, *p* < 0.001; 5‐HT6R^+/+^ versus 5‐HT6R^−/−^, *p* < 0.001. n = 3 from three mice in each group. Unpaired *t* test, data from 3 independent experiments. The data were normalized according to the SSTR3^+/+^ and 5‐HT6R^+/+^ groups. C‐D) There was a lower ratio of p‐CREB in SSTR3^−/−^ mice and 5‐HT6R^−/−^ mice compared to control mice. C. Immunohistochemistry results for p‐CREB in SSTR3^+/+^ and SSTR3^−/−^ mouse brain slices. p‐CREB (green) indicated activated CREB, and DAPI (blue) marked the nuclei. Scale bar, 20 µm. D. Immunohistochemistry results for p‐CREB in 5‐HT6R^+/+^ and 5‐HT6R^−/−^ mouse brain slices. p‐CREB (green) indicated activated CREB, and DAPI (blue) marked the nuclei. Scale bar, 20 µm. E‐F) The expression level of p‐CREB/T‐CREB was decreased in hippocampus of SSTR3^−/−^ and 5‐HT6R^−/−^ mice. E. Western blotting of p‐CREB and T‐CREB expression of hippocampus in SSTR3^−/−^ and 5‐HT6R^−/−^ mice, using β‐tubulin as a reference. For determination of the CREB phosphorylation levels in SSTR3^+/+^ and SSTR3^−/−^ mice, membranes were first incubated with p‐CREB antibody, and then stripped and incubated with T‐CREB antibody. F. Quantitative analysis of p‐CREB and T‐CREB expression ratio. SSTR3^+/+^ versus SSTR3^−/−^, *p* < 0.001; 5‐HT6R^+/+^ versus 5‐HT6R^−/−^, *p* < 0.001. n = 3 from three mice in each group. Unpaired *t* test, data from 3 independent experiments. The data were normalized according to the SSTR3^+/+^ and 5‐HT6R^+/+^ groups. G‐H) There was a significant decrease in the expression levels of 270 kDa and 480 kDa AnkG in hippocampus of SSTR3^−/−^ mice than in SSTR3^+/+^ mice. G. Western blotting of AnkG expression of hippocampus in SSTR3^+/+^ and SSTR3^−/−^ mice, using β‐tubulin as a reference. H. Quantitative analysis of AnkG different isoform expression. 190 kDa, *p* = 0.904; 270 kDa, *p* = 0.007; 480 kDa, *p* = 0.002. n = 3 from three mice in each group. Unpaired *t* test, data from 3 independent experiments. The data were normalized according to the SSTR3^+/+^ group. I) Four key binding sites for transcription regulator factor CREB to bind with the AnkG promoter region were predicted by the online website of JASPER. J) CREB cDNA increased the luciferase activity in HEK293T cells, while CREB inhibitor decreased it. CREB cDNA, *p* < 0.001; n = 9. 666‐15, *p* < 0.001; n = 9. Forskolin, *p* = 0.009, n = 9. Unpaired *t* test, data from 3 independent experiments. The data were normalized according to control group. The results were represented by mean ± SEM. K) A dominant negative form of CREB (KCREB) and CREB phosphorylation mutation (CREB^S133A^) both decreased the activity of AnkG promoter compared with CREB cDNA. One‐way ANOVA followed by Tukey post‐hoc test was based on data from three independent experiments; F _(3, 32)_ = 206.9; Promoter + CREB versus Promoter, *p* < 0.001; Promoter + CREB versus Promoter + KCREB, *p* < 0.001; Promoter + CREB versus Promoter + CREB^S133A^, *p* = 0.006. n = 9 for each group. The data were normalized according to AnkG‐promoter group. The results were represented by mean ± SEM. L) The combination of p‐CREB and AnkG promoter region was investigated by ChIP assay. Agarose gel electrophoresis for ChIP‐qPCR products. M) %Input of p‐CREB on AnkG promoter in ChIP‐qPCR. *p* < 0.001; n = 3 for each group. Unpaired *t* test, the results were represented by mean ± SEM. N) Comparison of AnkG promoter (promoter^1^ ^−802^ and promoter^802‐1902^) activity under basal conditions or CREB treatment. One‐way ANOVA followed by Tukey post‐hoc test was based on data from three independent experiments; F _(3, 32)_ = 531.6; promoter^1‐802^ versus promoter^802‐1902^, *p* < 0.001; promoter^1‐802^ versus promoter^1‐802^ + CREB, *p* < 0.001; promoter^802‐1902^ versus promoter^802‐1902^ + CREB, *p* = 0.001. n = 9 for each group. The data were normalized according to promoter^1‐802^ group. The results were represented by mean ± SEM. O) Schematic diagram showed four different mutation sites in the AnkG promoter and their relative locations. P) Four sites were target sequences of CREB on the AnkG promoter. Cells were transfected with the indicated plasmids, including four mutated firefly luciferase reporter gene plasmids as stated in (O) and CREB. Two‐way ANOVA followed by Sidak post‐hoc test; F _(4, 80)_ = 99.96; *p* < 0.001; n = 9 for each group. The data were normalized according to control AnkG‐promoter group. The results were represented by mean ± SEM.

The intersection in KEGG analysis prompted us to hypothesize that changes in PI3K‐Akt signaling might contribute to the changes in AIS. Previous studies have also shown PI3K‐Akt signaling is a central driver of cilia signaling‐mediated changes in axonal development and Akt is at the hub.^[^ ^18^ ^]^ Phosphorylated Akt (p‐Akt) is located at the basal region of the primary cilia.^[^ ^53^ ^]^ We examined the phosphorylation level of Akt and its transcription factor cyclic AMP‐response element binding protein (CREB) in control, SSTR3^−/−^ and 5‐HT6R^−/−^ mice. Immunoblotting revealed a decreased ratio of p‐Akt and total Akt (T‐Akt) in SSTR3^−/−^ and 5‐HT6R^−/−^ mice (**Figure** **8**A–B). Confocal microscopy revealed that phosphorylated CREB (p‐CREB) was attenuated in SSTR3^−/−^ and 5‐HT6R^−/−^ mice. (Figure 8C–D). There was also a decreased ratio of p‐CREB and total CREB (T‐CREB) in SSTR3^−/−^ and 5‐HT6R^−/−^ mice (Figure 8E–F). To explore the mechanisms underlying changes in AIS, we investigated AIS master organizer AnkG. In neurons, AnkG exists primarily in three isoforms: 190 kDa, 270 kDa, and 480 kDa. Among these isoforms, the 270 kDa and 480 kDa AnkG variants are predominantly localized at the AIS and the nodes of Ranvier.^[^ ^54^ ^]^ Shown by western blot, the expressions of 270 kDa and 480 kDa AnkG decreased in SSTR3^−/−^ mice (Figure 8G–H).

CREB plays a key role in promoting neuronal survival, precursor proliferation, neurite proliferation, and neuronal differentiation ^[^ ^55^ ^]^ and is involved in learning and memory.^[^ ^56^ ^]^ Previous studies have established CREB as a transcription factor responsible for regulating gene expression. To determine whether CREB regulates AnkG expression through transcriptional modulation, we first predicted the AnkG promoter region with the website JASPAR (http://jaspar.genereg.net/). There were 4 binding sites for CREB within 5′ upstream (2 kb) of the AnkG coding region (Figure 8I). Subsequently, we constructed a plasmid containing luciferase reporter gene under the control of the AnkG promoter sequence. The luciferase reporter assay indicated significantly higher luciferase activity when double‐transfected with CREB cDNA and the promoter plasmid compared to the control plasmid in HEK293T cells (Figure 8J). The CREB inhibitor 666‐15 reduced luciferase activity significantly, while agonist forskolin increased its activity (Figure 8J). Conversely, a dominant negative form of CREB (KCREB) which loses its transcriptional activity and mutation at Ser133 of CREB (CREB^S133A^) both distinctly decreased the activity of AnkG promoter compared with CREB cDNA (Figure 8K). The chromatin Immunoprecipitation (ChIP) assay results further revealed that CREB bound to the promoter region of AnkG (Figure 8L–M). Then, the luciferase activities of promoter^1^ ^−802^ and promoter^802‐1902^ were compared in the presence or absence of CREB. The relative luciferase activities increased in the promoter^802‐1902^ group compared to promoter^1^ ^−802^ in the absence or presence of CREB transfection, implying the existence of CREB‐binding sites at both the 0 to ‐802 and ‐802 to ‐1902 bp regions on the AnkG promoter (Figure 8N). To explore potential binding sites, four truncated mutants of the AnkG promoter, TGAAGTCA (‐1250‐1243), TGACCTGA (‐1142‐1135), TGATCTCA (‐597‐590), and TGATCTCA (‐24‐17) were obtained (Figure 8O). The relative luciferase activities of these four mutants were detected and analyzed by comparison with AnkG‐promoter. Under basal conditions (transfection with the empty CREB vector), the relative luciferase activity of each mutant was obviously lower than that of AnkG‐promoter. Similar results were obtained with CREB cDNA (Figure 8P). Therefore, we revealed these sites as novel target sequences of CREB on the AnkG promoter. These results implied that CREB extensively regulated AnkG expression by binding to multiple target sequences on the AnkG promoter.

### 2.6 CREB Activation Recovered AIS Structure and Function

**Figure 9:**
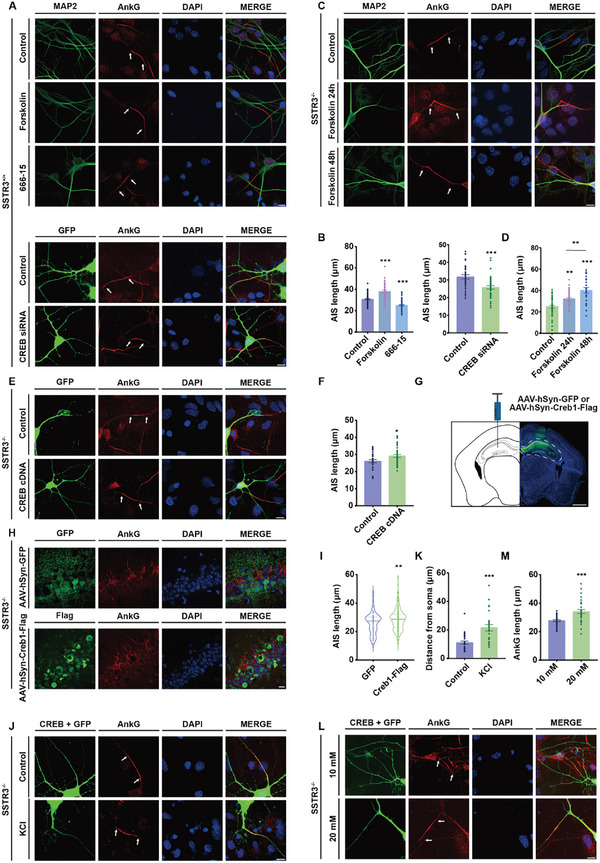
CREB expression restored AnkG structure and plasticity in SSTR3^−/−^ neurons. A‐B) Neurons in forskolin condition possessed longer AIS and AIS was shorter with 666‐15 and CREB siRNA treatments. A. Immunocytochemistry results for AnkG in the SSTR3^+/+^ neurons in control, forskolin, 666‐15 and CREB siRNA treatments. MAP2 and GFP (green) indicated morphology of neurons, AnkG (red) marked AIS, and DAPI (blue) marked the nuclei. Scale bar, 10 µm. B. Statistical graph of AIS length with different treatments. Left, One‐way ANOVA followed by Dunnet post‐hoc test was based on data from three independent experiments; F _(2, 146)_ = 55.51; control versus forsklin, *p* < 0.001; control versus 666‐15, *p* < 0.001. control, n = 53; forskolin, n = 38; 666‐15, n = 58. Right, Statistical graph of AIS length with CREB siRNA expression. *p* < 0.001; control, n = 35; CREB siRNA, n = 41. Unpaired *t* test, data from 3 independent experiments. The results were represented by mean ± SEM. C‐D) AIS length recovered in SSTR3^−/−^ neurons with forskolin treatments. C. Immunocytochemistry results for AnkG in the SSTR3^−/−^ neurons in control and forskolin condition. MAP2 (green) indicated dendrites and soma, AnkG (red) marked AIS, and DAPI (blue) marked the nuclei. Scale bar, 10 µm. D. Statistical graph of AIS length with forskolin treatments. One‐way ANOVA followed by Tukey post‐hoc test was based on data from three independent experiments; F _(2, 97)_ = 21.69; Control versus forskolin 24 h, *p* = 0.002; Control versus forskolin 48 h, *p* < 0.001; forskolin 24 h versus forskolin 48 h, *p* = 0.005. control, n = 43; forskolin 24 h, n = 33; forskolin 48 h, n = 24. The results were represented by mean ± SEM. E‐F) CREB cDNA expression restored AIS length in SSTR3^−/−^ neurons. E. Immunocytochemistry results for AnkG in the SSTR3^−/−^ neurons with control and CREB cDNA. GFP (green) indicated morphology of neurons, AnkG (red) marked AIS, and DAPI (blue) marked the nuclei. Scale bar, 10 µm. F. Statistical graph of AIS length with CREB cDNA expression. *p* = 0.017; control, n = 34; CREB cDNA, n = 40. Unpaired *t* test, data from 3 independent experiments. The results were represented by mean ± SEM. G) Schematic of stereotactic AAV injection and representative images showing AAV expression in the hippocampus. Scale bars, 1 mm. H‐I) CREB expression restored AIS length in brain slices. H. Immunostaining of CA1 region in mouse brain slices injected with AAV‐hSyn‐GFP or AAV‐hSyn‐Creb1‐Flag. AnkG (red) indicated the AIS region, and DAPI (blue) marked the nuclei. Scale bar, 10 µm. I. Statistical data of AIS length injected with AAV‐hSyn‐GFP or AAV‐hSyn‐Creb1‐Flag. *p* = 0.003; GFP, n = 95; Creb1‐Flag, n = 128. Unpaired *t* test, data from 3 independent experiments. J‐K) AIS position plasticity recovered with CREB cDNA expression in SSTR3^−/−^ neurons. J. Immunocytochemistry results for AnkG in CREB cDNA expression neurons with control and extracellular potassium (KCl) treatment. GFP (green) indicated morphology of neurons, AnkG (red) marked AIS, and DAPI (blue) marked the nuclei. Scale bar, 10 µm. K. Statistical graph of distance from soma. *p* < 0.001; control, n = 29; KCl, n = 25. Unpaired *t* test, data from 3 independent experiments. The results were represented by mean ± SEM. L‐M) AIS length plasticity recovered with CREB cDNA expression in SSTR3^−/−^ neurons. L. Immunocytochemistry results for AnkG in CREB cDNA expression neurons with control and glucose treatment. GFP (green) indicated morphology of neurons, AnkG (red) marked AIS, and DAPI (blue) marked the nuclei. Scale bar, 10 µm. M. Statistical graph of AnkG length in 10 mM and 20 mM glucose. *p* < 0.001; 10 mM, n = 43; 20 mM, n = 32. Unpaired *t* test, data from 3 independent experiments. The results were represented by mean ± SEM.

In our subsequent investigation, we aimed to elucidate the role of CREB in regulating the structure and function of AIS. SSTR3^+/+^ neurons were experimented with first. Treatment with forskolin induced a notable increase in AIS length, whereas treatment with the CREB inhibitor 666‐15 resulted in a reduction in AIS length (**Figure** **9**A–B). Similarly, treatment with CREB siRNA also led to a decrease in AIS length (Figure S5A–B, Supporting Information; Figure 9A–B). Furthermore, when SSTR3^+/+^ neurons were exposed to 666‐15, the proportion of single‐peak periodic structure of the AIS decreased (Figure S5C–D, Supporting Information). To explore the potential of CREB activation in restoring AIS structure, SSTR3^−/−^ neurons were treated with forskolin for 24 h and 48 h, resulting in a gradual increase in AIS length (Figure 9C–D). Additionally, introducing CREB cDNA into SSTR3^−/−^ neurons also led to a restoration of AIS length (Figure 9E–F). To identify whether CREB can regulate AIS length in the mouse brain, we performed stereotactic injection of AAV‐hSyn‐GFP or AAV‐hSyn‐Creb1‐IRES‐GFP in hippocampus (Figure 9G). As shown by immunohistochemistry, AIS became longer as the result of AAV‐mediated expression of CREB in hippocampus, a region known to be involved in learning and memory (Figure 9H–I). The structural plasticity of the AIS, which serves as the trigger zone of neurons, presents a potent mechanism for regulating neuronal activity.^[^ ^57^ ^]^ Following extracellular potassium treatment, the position of the AIS shifted away from the soma in SSTR3^−/−^ neurons with CREB cDNA (Figure 9J–K). The length of AIS increased in response to 20 mM glucose following CREB overexpression (Figure 9L–M). In summary, our findings indicated that replenishing CREB in SSTR3^−/−^ neurons restored both the structure and function of the AIS.

## 3 Discussions

The primary cilia and AIS are two sub‐cellular compartments with critical functions in neurons. While primary cilia serve as sensory organelles capable of detecting external signals and facilitating intracellular responses, the AIS integrates inputs from dendrites and the cell body to initiate APs.^[^ ^24^, ^58^ ^]^ Despite their spatial separation, our findings reveal an interaction between these two compartments.

Certain GPCRs are specifically localized only at the primary cilia. Among these, ciliary SSTR3 exhibits widespread expression throughout the brain, accounting for more than 90% of its total expression. Depletion of SSTR3 results in defects in object recognition memory.^[^ ^35^ ^]^ Furthermore, recent studies have demonstrated that mice with deletions in ciliary proteins within the hippocampus display various degrees of learning and memory deficits,^[^ ^12a^ ^]^ suggesting that ciliary proteins may play critical roles in cognition. Our results corroborate these findings, showing that knockout of SSTR3 leads to cognitive impairment in spatial cue‐related tasks, albeit not in working memory. We also observe that this behavioral deficit coincides with disruptions in synaptic function and neuron excitability. Upon closer examination on the single neuron, we observe significant changes in the AIS, a pivotal structure involved in APs initiation. Notably, AIS is also closely related to cognitive function. Mutations in AIS‐related genes are associated with various neurological diseases, and disruption in AIS is a common pathology in cognitive disorders.^[^ ^59^ ^]^ Altered AIS plasticity is involved in early pathogenesis in AD, as shown by AIS shortening in both AD mouse models and patients.^[^ ^60^ ^]^ Hemizygous AnkG knockout mice also exhibit cognitive impairments.^[^ ^61^ ^]^ Our observations provide an explanation for the impact of primary cilia alterations on neuronal excitability and neural circuit balance, offering new insights into the function of primary cilia in the nervous system. Due to the large molecular weight of 480 kDa, rescuing AnkG in adult SSTR3^−/−^ mice remains challenging. Further studies are required to clarify the specific role of the AIS in the memory deficits observed in SSTR3^−/−^ mice.

Previous studies have demonstrated that 5‐HT6R is located in the primary cilia, where it plays an important role in maintaining normal morphology and primary cilia function.^[^ ^44a^ ^]^ Overexpression of 5‐HT6R induces elongation and forks cilia morphology, while its deletion disrupts SHH signaling pathway.^[^ ^13c,d^ ^]^ 5‐HT6R also participates in neuron development, neurite growth and neural network formation.^[^ ^62^ ^]^ As such, it is often regarded as a therapeutic target for neurological diseases, including AD and schizophrenia.^[^ ^63^ ^]^ Recent research suggests that synapses form between serotonergic axons and 5‐HT6R‐expressing primary cilia of CA1 pyramidal neurons. Alterations in downstream signal within these cilia in mature neurons change nuclear actin, thus modulating hippocampal function by altering chromatin accessibility and transcriptional pathways.^[^ ^13e^ ^]^ Consistent with these findings, our results show that the elimination of 5‐HT6R affects intracellular signaling and alters the length of AIS. These observations provide a potential research direction for understanding pathological phenomena associated with abnormal 5‐HT6R expression.

IFT88 encodes a cilia‐specific protein essential for cilia formation, with its deficiency leading to a complete loss of the primary ciliary structure and disruption of ciliary signaling.^[^ ^64^ ^]^ In studies about primary cilia function, IFT88^LOX/LOX^ is commonly employed as a genetic model for ciliopathies.^[^ ^3^, ^65^ ^]^ Conditional deletion of IFT88 has been previously utilized to investigate primary cilia functions in a cell‐ or tissue‐specific context.^[^ ^66^ ^]^ Additionally, studies have identified the localization of IFT20 to the Golgi complex in mammalian cells, where it facilitates the transport of ciliary membrane proteins from the Golgi to the cilium. Other IFT polypeptides were not found in this pathway.^[^ ^67^ ^]^ Another study revealed that lymphoid and myeloid cells, which lack primary cilia, still express IFT proteins. IFT20 colocalized to a significant extent with the cis‐Golgi and coimmunoprecipitation experiments revealed the existence of an IFT20‐IFT88‐IFT57 complex.^[^ ^68^ ^]^ Kierszenbaum reported the localization of IFT88 in the Golgi apparatus of spermatids and its involvement in the development of the acrosome‐acroplaxome complex, the head‐tail coupling apparatus, and the spermatid tail. Spermatids from *Ift88* mutant mice exhibited abnormal head shaping and lack tails.^[^ ^69^ ^]^ Notably, sperm possess a flagellum, a modified cilium used for locomotion.^[^ ^70^ ^]^ Although flagellum and primary cilia differ in structure and function, both rely on the IFT system for protein transport.^[^ ^71^ ^]^ These findings suggest that the deletion of IFT proteins could impact organelles beyond primary cilia. However, given the structural and functional differences among cell types, it remains unclear whether IFT88 is localized in the Golgi apparatus of neurons.^[^ ^72^ ^]^ We label the cis‐Golgi using GM130 and assess its colocalization with IFT proteins in primary cultured neurons (Figure S5E–G, Supporting Information). Our results indicate strong colocalization between GM130 and IFT20, but minimal overlap with IFT88 (Figure S5E,F, Supporting Information). Concurrently, we analyze the colocalization of IFT88 with the ciliary marker ARL13B, finding substantial colocalization between IFT88 and ARL13B (Figure S5E,G, Supporting Information). IFT88 labels the bases of the primary cilia and shows weak punctate staining of the cilia. Further studies are required to more specifically characterize the role of Golgi apparatus in neurons lacking IFT88.

Electrophysiological studies of cultured hippocampal neurons in the “on‐cilia” configuration have revealed that ciliary ion channels can generate currents upon repolarization.^[^ ^10^ ^]^ Given that the primary cilia of hippocampal neurons extend from the soma, ion channels on these cilia may facilitate AP backpropagation from the axon to dendritic compartments.^[^ ^73^ ^]^ Our results show that alterations in primary cilia signaling also impact the structure of AIS where AP bursts occur. However, whether electrical conduction occurs within primary cilia and the extent of electrical coupling between primary ciliary compartment and AIS remain unknown. In the previous study, we combined voltage imaging with genetically encoded voltage indicators (GEVIs) and multicompartment electrophysiological modeling to map the dynamics of AP initiation and propagation in cultured hippocampal neurons.^[^ ^74^ ^]^ These methods can be applied in future studies to investigate the propagation characteristics of electrical signals within primary cilia.

Phosphorylated Akt has been identified at the basal region of primary cilia, supporting its role in ciliary function and signaling.^[^ ^53^ ^]^ Akt signaling within primary cilia has been shown to play a role across multiple cell types. Primary cilia in cholangiocytes contribute to tumor suppression through a PTEN‐Akt‐dependent mechanism.^[^ ^75^ ^]^ Ciliary elongation suppresses adipocyte differentiation via Akt downregulation.^[^ ^76^ ^]^ Moreover, primary cilia have been shown to modulate human decidualization through PTEN‐PI3K‐Akt‐FOXO1 signaling.^[^ ^77^ ^]^ In hippocampus neurons, Akt mediates cilia shortening in cells expressing melanin‐concentrating hormone receptor 1, indicating that Akt signaling is closely linked to primary cilia in neurons.^[^ ^78^ ^]^ A recent study has further demonstrated that cilia‐localized insulin‐like growth factor 1 receptor and downstream Akt signaling are essential for the neuroprotective function of cilia in cortical neurons. The loss of primary cilia can disrupt dendritic arborization, potentially due to defective cilia and downstream Akt signaling.^[^ ^79^ ^]^ In this study, we also find that signaling defects in primary cilia alter the Akt pathway, impacting downstream biological processes, including AnkG transcription. These findings support the idea that dysregulated ciliary signaling can propagate throughout the whole neuronal cell body to manifest pathogenic changes in normal state. This pathway may explain how primary cilia influence neuronal excitability and neural circuit balance by altering AIS.

Primary cilia signaling is likely to influence neuronal behavior via both rapid local signaling cascade changes as well as by triggering changes in transcriptional programs. Our results show that ciliary GPCR SSTR3 activation induces CREB phosphorylation and regulates protein expression of key AIS “hub” AnkG via transcriptional modulation. CREB, a ubiquitous transcription factor, plays a multifaceted role in various cellular processes, including proliferation, survival, differentiation, and neuronal excitability.^[^ ^80^ ^]^ Increasing CREB function increases the propensity of neurons to burst APs, while decreasing CREB function decreases neuronal excitability.^[^ ^81^ ^]^ Our study proposes that CREB may serve as a transcriptional regulator of AnkG. As a critical scaffold protein, AnkG expression level influences the clustering of ion channels within the AIS, potentially elucidating the mechanism by which CREB participates in the regulation of neuronal excitability. Notably, it has previously been demonstrated that regulating the cAMP pathway rescues dendritic abnormalities in AnkG deficiency models ^[^ ^82^ ^]^ and absence of AnkG alters behavioral responses induced by forskolin.^[^ ^83^ ^]^ The restricted spatial structure of the cilia allows for rapid diffusion of signal molecules. Thus, we hypothesize that primary cilia may serve as pivotal structures for signal transmission, with their response to external stimuli altering neuronal structure to modulate signal output.

The expression of AnkG shows tissue‐ and cell‐specific variation, and encodes multiple protein isoforms. There are several transcription starting sites for the rodent *Ank3* gene. AnkG isoform expression may be regulated not only through alternative splicing but also through alternative start sites. In addition, the transcriptional activities of different exons of *Ank3* in different brain regions are also significantly different. It remains unclear whether transcription initiation at different exon promoters determines downstream splicing regulation of exon 37.^[^ ^84^ ^]^ Here we focus on the transcription start site upstream of exon 1e, expressed in many tissues including the frontal cortex, hippocampus, and caudate putamen ^[^ ^85^ ^]^ and point out the expression of 270 kDa and 480 kDa AnkG is decreased. Similarly, the 5′bipolar disorder risk allele overlaps two first exons of *Ank3* (exon1b and exon1e) ^[^ ^54^, ^85^ ^]^ and their associated promoters.

AIS continuously adapts to its surrounding environment, with changes occurring on timescales from milliseconds to days.^[^ ^86^ ^]^ During critical periods of early embryonic development, the AIS undergoes substantial structural transformations that align with significant shifts in neuronal excitability.^[^ ^87^ ^]^ In mature neurons, AIS morphology is also influenced by neuronal activity. For example, hyperactivity in excitatory neurons leads to structural changes in AIS and decreases excitability, maintaining activity within an optimal range.^[^ ^88^ ^]^ In our study, we adopt the methods of Grubb and Wang, using chronic depolarization with high extracellular potassium to induce AIS position plasticity without affecting its length, while AIS length plasticity is only induced following high glucose treatment.^[^ ^48^, ^88^ ^]^ The activity‐dependent structural plasticity is characterized by the concomitant relocation of cytoskeleton elements, such as AnkG. Notably, we find that knocking out the ciliary protein SSTR3 abolishes AIS plasticity, likely related to reduced AnkG expression in SSTR3^−/−^ mice.

In summary, we report that the morphology and function of the primary cilia change with SSTR3 ablation. It is interesting that changes in primary cilia can lead to abnormalities in AIS structure and function through CREB signaling and regulation of AIS scaffolding protein AnkG. It might be a novel mechanism by which ciliary signaling may modulate neuronal activity, such as AP firing and propagation through the AIS. Our results provide new insights of the function of primary cilia in the nervous system.

## 4 Experimental Section

### Animals

SSTR3^−/−^ mice were purchased from The Jackson Laboratory (B6.129S4(129S6)‐Sstr3^1Ute^/J, #0 08123).^[^ ^89^ ^]^ IFT88^LOXP/LOXP^ mice were generously provided by Dr. Yi Rao at Peking University, which were originally purchased from The Jackson Laboratory (B6.129P2‐Ift88^1Bky^/J, #02 2409).^[^ ^90^ ^]^ AnkG^LOXP/LOXP^ mice were generously provided by Dr. Vann Bennett.^[^ ^91^ ^]^ The development of the Htr6 gene‐modified mouse model (Htr6‐creERT2‐P2A‐EGFP‐SV40PA) was carried out in strict accordance with the guidelines of the Institutional Animal Care and Use Committee (IACUC) at the Shanghai Model Organism Center, Inc (2015‐0003‐903). All mice were maintained in compliance with the Animal Care and Use guidelines of Peking University (LSC‐ZhangY‐1).

### Plasmids and siRNAs

pEGFPN3‐Sstr3 was a gift from Kirk Mykytyn (Addgene plasmid # 35 623). Cilia‐GECO1.0 (5HT6‐mCherry‐G‐GECO1.0) was a gift from Takanari Inoue (Addgene plasmid # 47 500). Dr. Kirk Mykytyn (Ohio State University, USA) generously provided the mouse 5‐HT6R plasmid. Mouse mCherry‐tagged IFT88 was amplified from cDNA from the mouse brain. mCherry‐N3 was purchased from Addgene. The plasmid of CREB cDNA was obtained from GENECHEM (China), and Cre‐BFP was from OBiO Technology (China). CREB dominant‐negative vector set was from Clontech. SSTR3‐siRNA was purchased from Qiagen. CREB siRNA (sc‐35111, Santa Cruz Biotechnology) was used to knock down SSTR3. SSTR1‐sgRNA and SSTR2‐sgRNA were from Beyotime.

### Primary Neuron Culture and Transfection

Primary hippocampal neurons were cultured from postnatal day 0 mouse pups. The hippocampal tissues underwent enzymatic digestion using 0.25% trypsin (Solarbio) for a 15‐minute incubation at 37 °C. An equal volume of DMEM/F‐12 (Gibco) supplemented with 10% fetal bovine serum (FBS) (Gibco) was added to stop the reaction. Each cell suspension was gently dispersed by blowing, followed by a 2‐minute centrifugation step at 500 ×g to facilitate cellular sedimentation. Subsequently, neurons were resuspended in DMEM/F‐12 with 10% FBS and plated on poly‐D‐lysine‐coated coverslips for 30 min. After half an hour, Neurobasal medium (Gibco) containing 1% Pen‐Strep (Invitrogen), B27 (Gibco), and GlutaMAX (Thermo Fisher) was added to the medium. For glucose treatment, Neurobasal‐A medium (Gibco) containing Pen‐Strep, B27, and GlutaMAX supplemented with 10 mM D‐glucose (Sigma) was used. Half of the medium was replaced with fresh medium every 3–4 days. The cells were maintained in a 37 °C incubator of 5% CO_2_.

For neurons, transfection was conducted using a calcium phosphate precipitation method between 5–8 days post‐culture. A mixture containing DNA, CaCl_2_, 2 × HEPES‐buffered saline (HBS), and sterile H_2_O was carefully added to each well, permitting a 1–2 hours incubation period. Then, 1 × HBS (pH 6.8) was added to each well to remove the precipitate. Thereafter, the original medium was reintroduced to the wells.

### Cell Lines Culture and Transfection

For HEK293T cells, the cryotube containing cells were transitioned from liquid nitrogen to a 37 °C water bath. DMEM medium enriched with 10% FBS was added. Following a brief centrifugation step lasting 2 min at a rate of 500 × g, the supernatant was discarded and the cell precipitates were resuspended with DMEM medium containing 10% FBS. The cell suspension was plated on coverslips. DMEM medium enriched with 10% FBS and 1% GlutaMAX was used for BV2. DMEM/F‐12 with 10% FBS was used for hippocampal neuronal cell line HT22.

For cell lines transfection, 1 µg target plasmid was added into 35 µL Opti‐MEM medium and 3 µL PEI (3 times the volume of the target plasmid) was also added to 35 µL Opti‐MEM medium for one of the 24‐well plates. Then two solutions were mixed and incubated for 30 min at 37 °C. The mixture was dripped into each well. After 6 h, the medium was replaced with DMEM medium with 10% FBS.

### Calcium Imaging In Vitro

For the calcium imaging experiments of primary cilia, we referred to the previous studies and modified it.^[^ ^8^ ^]^ Neurons were transfected with Cilia‐GECO1.0 and imaged at an acquisition rate of 0.5 Hz. Cells were maintained in artificial cerebrospinal fluid (ACSF) containing 2 mM Ca^2+^. The effects of ATP were assessed by recording for 1 min before and 4 min after addition. GECO1.0 fluorescence values subtracted background and then divided by the mCherry values measured. They were normalized against the average baseline values prior to ATP addition.

### Immunostaining

Cells were usually fixed with 4% paraformaldehyde (PFA) for 20 minutes at room temperature. Cold methanol was used for immunostaining of primary cilia at ‐20 °C. Then cells were permeabilized with 0.1% Triton X‐100 in PBST (0.1% Tween‐20 in PBS) for 15 min at 4 °C and washed three times with PBST. Fixed neurons were blocked with 5% bovine serum albumin (BSA) in PBST for one hour at room temperature. Neurons were incubated with primary antibodies overnight at 4 °C. On the next day, neurons were incubated for 1 h at room temperature with Alexa Fluor‐conjugated secondary antibodies (1:500, Invitrogen). The nuclei were stained with 4′,6‐diamidino‐2‐phenylindole (DAPI, Sigma) for 10 min, after which they were transferred to slides for imaging.

Primary antibodies included rabbit anti‐AnkG (1:200, sc‐12719, Santa Cruz Biotechnology), rabbit anti‐NF186 (1:1000, ab31457, Abcam), mouse anti‐β‐IV‐spectrin (1:500, N393/76, NeuroMab), mouse anti‐Na_v_1.2 (1:500, K69/3, NeuroMab), rabbit anti‐adenylyl cyclase 3 (1:2000, C‐20, Santa Cruz Biotechnology), goat anti‐SSTR3 (1:1000, M‐18, Santa Cruz Biotechnology), mouse anti‐ARL13B(N295B/66) (1:1000, 75–287, NeuroMab), rabbit anti‐5‐HT6R (1:1000, ab103016, Abcam), rabbit anti‐NeuN (1:1000, 702 022, Invitrogen), chicken anti‐MAP2 (1:10 000, ab5392, Abcam), rabbit anti‐IFT20 (1:1000, 13615‐1‐AP, Proteintech), rabbit anti‐IFT88 (1:1000, 13967‐1‐AP, Proteintech) and mouse anti‐GM130 (1:1000, 66662‐1‐lg, Proteintech).

### Immunohistochemistry

The mice were anesthetized and perfused with saline solution and 4% PFA solution. The mouse brains were extracted and incubated in 4% PFA solution at 4 °C overnight. After fixation, the brains were successively dehydrated in saline solution containing 20% and 30% sucrose. The dehydrated brain tissues were embedded in O.C.T. (4583, Sakura Finetek) and frozen at ‐80 °C. The frozen brain tissues were cut into 10–20 µm coronal sections. After washing with PBS, the sections underwent transmembrane treatment using PBS containing 0.1% Tween‐20 and 0.3% Triton X‐100 for 15 minutes at room temperature. Subsequently, the sections were blocked with 5% BSA in PBST followed by incubation with primary antibody overnight at 4 °C. Sections were extensively washed in PBST, and then incubated with secondary antibodies (1:500, Invitrogen) for 2 hours at room temperature. The nuclei were stained with DAPI (Sigma) for 15 min.

Primary antibody: goat anti‐SSTR3 (1:1000, M‐18, Santa Cruz Biotechnology), rabbit anti‐AC3 (1:2000, C‐20, Santa Cruz Biotechnology, Santa Cruz, CA), rabbit anti‐AC3 (1:10 000, RPCA‐ACIII, Encor), mouse anti‐ARL13B (N295B/66) (1:500, 75–287, NeuroMab), mouse anti‐Flag (1:1000, M20008, Abmart), rabbit anti‐AnkG (1:500, OM167212, OmnimAbs) and rabbit anti‐p‐CREB (1:1000, 9198, Cell Signaling Technology).

### Morris Water Maze Test

Morris water maze test was performed as previously described.^[^ ^13d^ ^]^ Briefly, the mice underwent training and were recorded in Morris water maze for 6 consecutive days to measure their swimming speed, the time taken to reach the platform, and the swimming distance to the hidden platform. On the seventh day, the hidden platform was removed and the time spent in the target quadrant without the platform was documented, along with the number of crossings at the original location of the platform. The Morris water maze consisted of a circular water pool measuring 160 cm in diameter and 50 cm in height, with a hidden platform positioned beneath the water surface. Initially, mice were allowed to swim freely for 90 seconds without the platform to acclimate to the water environment (day 0). From day 1 to day 6, the test began when a mouse was placed in one of the quadrants facing the wall and ended when the mouse reached the platform. If the mouse successfully reached the platform within 90 s, it was permitted to remain on the platform for an additional 10 s. In cases where the mouse failed to locate the platform within 90 s, it was gently guided to the platform and allowed to stay for 10 s. Real‐time dynamic tracking and data acquisition were conducted by an automatic tracking system.

### Y‐Maze Test

Y‐maze test was performed as previously described.^[^ ^13^, ^92^ ^]^ The equipment used for this experiment consisted of three arms, each measuring 34 cm in length, 8 cm in width, and 14.5 cm in height. At the ends of the arms, three distinct images were displayed: a red circle, a black square, and a white triangle. Mice were placed on the apparatus facing the wall at the beginning. The observation period lasted for 10 min. During this time, an automatic tracking system monitored the position of the mouse in real‐time and recorded the number of times the mouse visited a new arm. The arm was considered new when it differed from both the preceding and subsequent arms chosen by the mouse.

### Rotarod Test

A rotating rod (ENV‐575A, Med Associates) was set to spin at 4 rpm, with a lane width of 50 mm and a diameter of 30 mm. On the first day, the rotating rod was maintained at 4 rpm to allow each mouse to adapt for 5 minutes. On the next day, the mice were subjected to an increase in speed from 4 to 40 rpm. Each mouse underwent three trials with a 60‐minute interval. On the test day, the rotation speed of the rotating rod was increased from 4 to 40 rpm, and each mouse was tested in three trials with a 60‐minute interval. The time that the mice remained on the rod and the velocity at which they fell off were recorded on the test day. The data from three trials were averaged for analysis.

### Biocytin Labeling and Immunostaining

Coronal sections 300 µm thick were recorded using patch clamp under whole‐cell configuration. During whole‐cell recording, the neuronal spine morphology of CA1 neurons was labeled by adding 1% biocytin (B4261, Sigma) to the pipette solution. Only neurons that maintained stable membrane potential for at least 20 min were included. On cessation of filling, the pipette was slowly pulled out along the direction of recording until a membrane reseal was formed. After recording, slices were fixed overnight in 4% PFA, followed by permeation of 0.3% Triton X‐100 for 30 min and incubation with streptavidin‐coupled Alexa 488 (S11223, Invitrogen) for 2 h at room temperature.

### Spine Acquisition and Analysis

For spine morphology analyses, z‐stacks of secondary dendritic stretches were captured using a 60× oil‐immersion objective on a laser scanning confocal microscope (Nikon AXR laser scanning microscope). In biocytin‐labeled samples, spines were imaged in 20 µm z‐stacks with a 0.2 µm z‐step. For spare‐labeling, spines were captured in 10 µm z‐stacks, also with a 0.2 µm z‐step. Spines were then automatically reconstructed and manually adjusted using the Filament Tracer module of Imaris. Dendritic spines were classified into three classes using the “Imaris Spines Classifier” extension in the Imaris software with the following morphological criteria:^[^ ^93^ ^]^ (1) mushroom spines had a head diameter larger than 0.5 µm and a head‐to‐neck diameter ratio greater than 1.1; (2) long thin spines included a head‐to‐neck diameter ratio less than 1.1 and a length‐to‐spine head diameter greater than 2.0; (3) Stubby spines were the remaining spines. All steps were carried out blindly to the experimental conditions.

### Electrophysiological Recordings

To evaluate neuronal excitability, whole‐cell patch‐clamp recordings were conducted on cultured hippocampal neurons. The patch pipette had a resistance ranging from 3 to 5 MΩ. The extracellular solution was composed of AP5 (25 µM), GABAzine (20 µM), and 2,3‐dihydroxy‐6‐nitroaminosulfamoylbenzo (6‐nitro‐7‐sulfamoylbenzo(f) quinoxalin‐2,3‐dione, NBQX) (10 µM). The patch pipette was carefully lowered until it made contact with the neuron cell body. Upon achieving a high‐resistance seal, the recording mode was switched to voltage clamp mode, with a holding potential set at ‐60 mV. Current stimulation was administered with a gradient of 10 pA. The MultiClamp 700B patch‐clamp amplifier and Digidata 1440A data acquisition system with Clampex software were used for data acquisition. The data was analyzed using Clampfit software.

For LTP recording, the mice were anesthetized, placed on ice, and injected with ACSF to maintain neuronal activity. After perfusion, the whole brain tissue was extracted and incubated for 2 min in an acute brain slice incubator. Subsequently, the brain was quickly transferred to a microtome and cut into brain slices with a thickness of 400 µm. focusing on the hippocampal region. These slices were then immersed in oxygenated ACSF and subjected to a 40‐minute incubation at room temperature, followed by a 30‐minute incubation in a new ACSF at 34.3 °C. LTP recording used dual probe resistance‐feedback high speed current clamp amplifiers MultiClamp 700B and Clampex 10.6. The stimulation electrode and the recording electrode were lowered to the corresponding positions. The stimulation intensity employed for LTP was set at 50% intensity obtained during input‐output (I‐O) curve recording. To establish a statistical baseline, fEPSPs induced for 20 min were recorded. After the baseline recording, a brief period of high‐frequency stimulation was introduced, after which the recording continued for 65 min. Statistical results were normalized relative to the baseline.

### Golgi Staining

Golgi staining was performed with the Hito Golgi‐Cox OptimStain Kit (HTKNS1125, HitoBiotec Corp). A modified method was used according to the method described in the instructions. The specific steps were as follows: After the mouse was anesthetized, the complete brain was delicately extracted. The brain was transferred to the impregnation solution and incubated at room temperature in the dark for 12–24 h. The next day, the impregnation solution was replaced and the brain was incubated at room temperature for 2 weeks. The brain tissue was transferred to Solution 3 and stored at 4 °C for 24–72 h. The tissue was coated in low gelling temperature agarose and cut into 200 µm sections. The slices were rinsed twice in distilled water for 3 min and then immersed in a mixture of Solution 4 and Solution 5 for 10 min for staining. After two additional rinses, the brain slices were dehydrated in ethanol solutions of increasing concentrations (50%, 75%, 95%, and 100%) for 5 min each. Finally, the brain slices were dehydrated in 100% ethanol for 3 times for 5 min each time, followed by treatment with xylene to make them transparent for 5 min each time. Neutral resin was used on coverslips.

Dendritic spines were divided into three categories according to the length of their “neck” and the width of their “head”. Dendritic spines with enlarged heads, widths greater than 0.65 µm, and neck lengths greater than 0.65 µm were referred to “mushroom”. Those with head widths less than 0.65 µm and neck lengths exceeding 0.65 µm were termed as long dendritic spines (thin), while those with neck lengths less than 0.65 µm were designated as stubby.^[^ ^39b^ ^]^

A deep learning approach was employed to identify and quantify dendritic spine types. Dendritic spines were classified and counted using a Mask Region‐based Convolutional Neural Network (Mask R‐CNN).^[^ ^94^ ^]^ This process involved a two‐stage approach: first, a region proposal network identified candidate regions, followed by classification, regression, and pixel‐level segmentation. The Mask R‐CNN algorithm was used to train a model for detecting neuronal dendritic spines in brain slices, with spine type prediction and analysis conducted at a 50% confidence threshold. This confidence interval aligned with conventional prediction model requirements.

### Primary Cilia and AIS Measurement

Confocal microscopy was employed to assess the length of both primary cilia and the AIS. Samples were imaged on Live SR CSU W1 (Nikon TiE) equipped with a 60×/1.27 objective and a 100×/1.4 Plan‐Apochromat objective. Lasers with wavelengths of 405, 488, 561, and 633 nm were used for excitation. Confocal z‐stacks were recorded with z‐step size of 0.2 µm. The images were processed identically and used Fiji software to generate maximal projections. All depicted images showed a maximum projection of a z‐stack unless differently stated in the figure legend. The length of the cilia was determined by measuring the ciliary protein signals using z‐stacked max‐signal intensity projected images in ImageJ. For statistical analyses of ciliary length, the three experimental replicates were combined and represented all data as scatter plots. For AIS measurement, a line profile was drawn starting at the soma that extended down the axon, through and past the AIS. The “start” and “end” positions were defined as the proximal and distal axonal points, respectively, at which the profile dipped to 33% of its peak value.^[^ ^88b^ ^]^

### Super‐Resolution Imaging and Analysis

A 3D SIM (DeltaVision, OMX) was used along with commercial software for imaging and 3D SIM reconstruction. The 3D SIM system equipped with a 60× oil immersion lens (NA: 1.42, Olympus) and a camera with a pixel size of 4.8 µm. Super resolution imaging method was referred to the previous article.^[^ ^48b^ ^]^ The reconstructed images were projected by maximum intensity, and Fiji software was employed to select the region of interest (ROI). Then the ROI region was summed up in the vertical direction, the space between the two peaks was quantified. In conjunction with the pixel size of the images, a histogram analysis was conducted. Unimodal fitting: unimodal fitting. Bimodal Gaussian distribution: R^2^ < 0.85 for the unimodal fitting and R^2^ ≥ 0.80 for the bimodal fitting. Nonperiodic pattern: R^2^ < 0.85 for the unimodal fitting and R^2^ < 0.80 for the bimodal fitting.

### Western Blotting

Tissue samples were collected with RIPA lysis buffer (R&D). Protein was obtained by centrifugation at 16 000 rpm for 30 min at 4 °C. Subsequently, sodium dodecyl sulfate (SDS) was added to the protein sample and heated at 95 °C for 10 min. The boiled protein was stored at ‐80 °C for subsequent experiments.

The samples and protein markers were separated into 10% sodium dodecyl sulfate‐polyacrylamide gel electrophoresis (SDS‐PAGE) at 80 V for 30 min and 160 V for 40 min. Polyvinylidene difluoride (PVDF) membranes (Millipore) were cut to appropriate size and activated in anhydrous methanol. The protein was transferred to PVDF membrane at 25 mV for 50 min. Once transferred, the membranes were blocked in 5% BSA (Sigma) in Tris‐buffered saline (TBS, Sigma) with 0.1% Tween 20 (TBST) at room temperature for 1 h. Primary antibodies were incubated overnight at 4 °C. The following day, after washing three times for 5 min each with TBST, horseradish peroxidase (HRP)‐conjugated secondary antibodies were added. Enhanced chemiluminescence was used to detect the optical density of HRP. BioRad ChemiDox (BioRad) was used to analyze the optical density of HRP. For determination of the CREB phosphorylation levels, membranes were first incubated with p‐CREB antibody, and then stripped by stripping buffer (AQ540, Analysis Quiz) and incubated with T‐CREB antibody.

Primary antibodies were used including goat anti‐SSTR3 (1:1000, M‐18; Santa Cruz Biotechnology), rabbit anti‐p‐Akt (Ser 473) (1:1000, 4060, Cell Signaling Technology), rabbit anti‐Akt (pan) (C67E7) (1:1000, 4691, Cell Signaling Technology), rabbit anti‐p‐CREB (1:1000, 9198, Cell Signaling Technology), rabbit anti‐T‐CREB (1:1000, A10826, ABclonal), rabbit anti‐AnkG (1:1000, OM167212, OmnimAbs), rabbit anti‐Gli1 (1:1000, ab151796, Abcam), rabbit anti‐Gli2 (1:1000, ab167389, Abcam), rabbit anti‐Gli3 (1:1000, ab69838, Abcam), rabbit anti‐SSTR1 (1:1000, 20587‐1‐AP, Proteintech) and mouse anti‐SSTR2 (1:1000, sc‐365502, Santa Cruz Biotechnology).

### RNA Isolation, Sequencing, and Data Analysis

Total RNA was extracted from freshly dissected tissues using the TRNzol Reagent kit (DP405, Tiangen) according to the manufacturer's instructions. RNA was assessed using the Agilent 2100 BioAnalyzer (Agilent Technologies) and Qubit Fluorometer (Invitrogen), ensuring an RNA integrity number (RIN) > 7.0 and a 28 S:18 S ratio > 1.8. Three biological replicates from each group were evaluated in this manner. RNA‐seq libraries were generated and sequenced by CapitalBio Technology. The NEB Next Ultra RNA Library Prep Kit for Illumina (NEB) was employed for library construction. The NEBNext Poly(A) mRNA Magnetic Isolation Module (NEB) kit was used to enrich poly(A)‐tailed mRNA molecules from 1 µg of total RNA. The mRNA was fragmented into ≈200 bp pieces. First‐strand cDNA was synthesized from the mRNA fragments using reverse transcriptase and random hexamer primers, while second‐strand cDNA synthesis was performed with DNA polymerase I and RNase H. The cDNA fragments were then end‐repaired, including the addition of a single “A” base, followed by adapter ligation. The products were purified and amplified via polymerase chain reaction (PCR) to construct the library DNA. Library quantification was carried out using the KAPA Library Quantification kit (KAPA Biosystems) and the Agilent 2100 Bioanalyzer. After quantitative reverse transcription‐polymerase chain reaction validation, libraries were subjected to paired‐end sequencing with 150‐base pair read lengths on an Illumina NovaSeq sequencer (Illumina). The human genome version of hg19 was used as a reference. The sequencing quality was assessed with FastQC (Version 0.11.5),^[^ ^95^ ^]^ and data with large noise were filtered using NGSQC (v0.4).^[^ ^96^ ^]^ The clean reads were aligned to the reference genome using HISAT2 (version 2.1.0) (Johns Hopkins University, USA).^[^ ^97^ ^]^

Gene expression analysis was performed using StringTie (v1.3.3b),^[^ ^98^ ^]^ and differential gene expression was assessed with DESeq (v1.28.0).^[^ ^99^ ^]^ *P*‐values were corrected by false discovery rate (FDR), and corrected *p*‐values (*q*‐values) were calculated by correcting using Benjamini–Hochberg (BH) method. Both *p*‐values and *q*‐values were used for significance analysis, with significantly differentially expressed genes defined as those with *p* ≤ 0.05. The annotations of the DEGs were performed based on the information obtained from the database of ENSEMBL, NCBI, Uniprot, GO, and KEGG.^[^ ^100^ ^]^ Heatmap was plotted by https://www.bioinformatics.com.cn, an online platform for data analysis and visualization.^[^ ^101^ ^]^ The RNA‐seq datasets have been deposited in Gene Expression Omnibus (GEO): GSE248779.

### Luciferase Reporter Assay

Plasmids of pGL4.20 [luc2/Puro] vector and pGL4.47 [hRluc/TK] vector were obtained from Promega. HEK293T cells were seeded in 6‐well plates. After 24 h post‐transfection, cells were washed with PBS and lysed, and luminescence was measured following instruction of Dual‐luciferase Reporter Assay System (Promega). CREB inhibitor 666–15 (100 nM, Selleckchem) and forskolin (10 µM, 93 049, sigma) were used.

### ChIP Assay

BV2 cells were crossing‐linked by 37% PFA at room temperature for 5 min and quenched with glycine to a final concentration of 125 mM. Cells were then lysed by ChIP lysis buffer and sonicated. Pre‐cleared chromatin supernatants were subjected to immunoprecipitation at 4 °C overnight using ChIP‐grade antibodies against p‐CREB (9198, Cell Signaling Technology). As a negative control, an equal amount of chromatin was precipitated with rabbit IgG antibody (2729, Cell Signaling Technology). Antibody‐chromatin complexes were then precipitated by ChIP‐Grade Protein G Magnetic Beads (9006, Cell Signaling Technology) for 3 h at 4 °C. DNA samples were eluted and reversed cross‐linking. Purified DNA was subjected to qPCR using TB Green Premix Ex Taq (RR420A, Takara). Forward and reverse primers were designed to amplify the AnkG promoter region containing one predicted binding site for CREB. After amplification, PCR products were resolved on a 2% agarose gel and visualized by ethidium bromide staining.

### Stereotaxic AAV Injection

For stereotaxic AAV injection, mice were anaesthetized and placed in a stereotaxic frame, and small craniotomies were made over the target brain regions. Stereotaxic coordinates of virus injection were as follows: AP −2.00 mm, ML 1.80 mm, DV −1.45 mm. To capture the dendritic spine density of individual neurons, we employed sparse labeling based on the recombinase system‐dependent co‐packaging strategy (n = 3 each group). The AAV‐sparse‐NCSP‐YFP‐2E5 (5.45 × 10^12^ vg mL^−1^) virus was purchased from Braincase Co., Ltd. (Shenzhen, China). Glass micropipettes preloaded with AAV‐sparse‐NCSP‐YFP‐2E5 (200 nL) were gradually lowered into mice to deliver the virus at a rate of 20 nL min^−1^. The microsyringe was kept in place for 10 min to prevent virus backflow upon withdrawal. Follow‐up experiments were performed after 2 weeks of AAV injection. Each mouse was injected with 6 × 10^10^ vg AAV for CREB overexpression. AAV9‐hSyn‐GFP (3.21 × 10^13^ vg mL^−1^) and AAV9‐hSyn‐Creb1‐Flag‐IRES‐GFP (4.10 × 10^13^ vg mL^−1^) were packaged at Vigene Biosciences. Follow‐up experiments were performed after 2 weeks of AAV injection.

### Statistical Analysis

Data were collected and analyzed using GraphPad Prism 8.0 software with an alpha value set at 0.05 to indicate statistical significance. The results were represented by the mean ± SEM for bar charts. For violin plots, the dashed lines represent the median (black dashes) and the interquartile range (colored dashes). Details of biological replicates and statistical analysis were described in the corresponding figure legends. Two‐group comparisons were analyzed using the two‐tailed Student's t‐test. Comparisons among multiple groups with one independent factor were done with ordinary one‐way ANOVA followed by post hoc analysis with Dunnett's multiple comparison test or Tukey's multiple comparison test. Comparisons among multiple groups with two independent factors were performed using two‐way ANOVA followed by post hoc analysis with Sidak multiple comparison test. In all cases, significance was defined as: *p < 0.05, **p < 0.01, and ***p < 0.001. And ns was considered nonsignificant (*p* > 0.05).

## Conflict of Interest

The authors declare no conflict of interest.

## Author Contributions

H.W., Y.L., and X.L. contributed equally to this work. H.W., Y.L., X.L., and Z.S. performed all experiments and analyzed the data. F.Y., A.P., D.K., H.W.L., H.C., and H.H. helped in modeling, cellular assays, animal behavioral tests, neuronal culture, project design, and manuscript drafting. Y.Z. conceptualized the study, performed analyses, and drafted the manuscript with inputs from all authors. All authors have read and approved the final manuscript.

## Supporting information



Supporting Information

## Data Availability

The data that support the findings of this study are available on request from the corresponding author. The data are not publicly available due to privacy or ethical restrictions.
