# Supplementary material for: The Primary Cilia are Associated with the Axon Initial Segment in Neurons
Source: Adv Sci (Weinh). 2025 Jan 13;12(9):2407405. doi: 10.1002/advs.202407405 (PMC11884599; doi:10.1002/advs.202407405)
Supplement: Supplementary file 1 — Supporting Information [file ADVS-12-2407405-s001.docx]

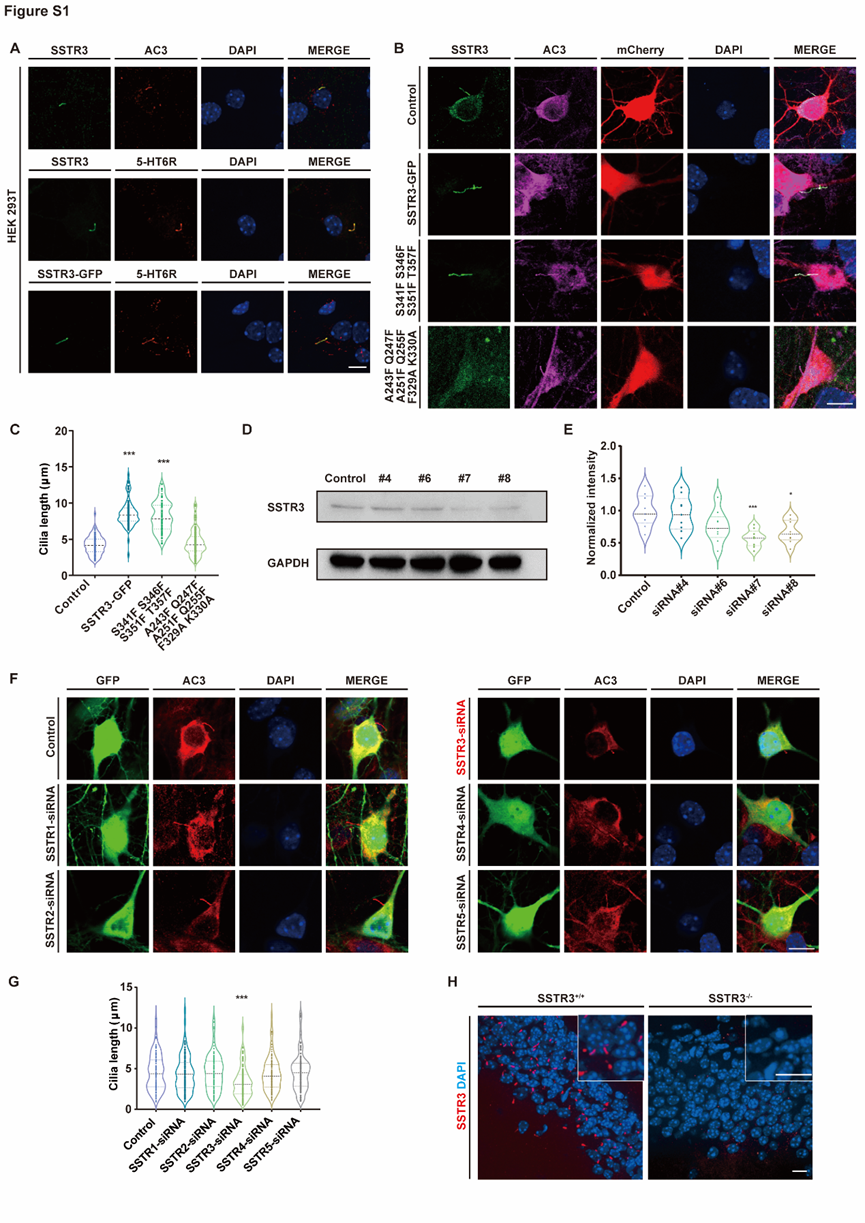


**Figure S1.** **Disruption of SSTR3 resulted in changes in cilia length.**


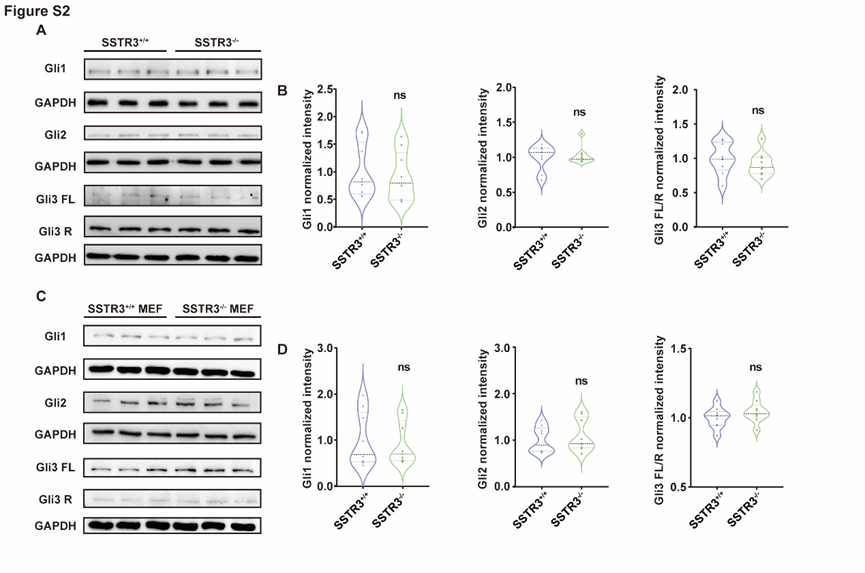


**Figure S2. SSTR3 deletion did not affect the normal expression of SHH signaling pathway.**


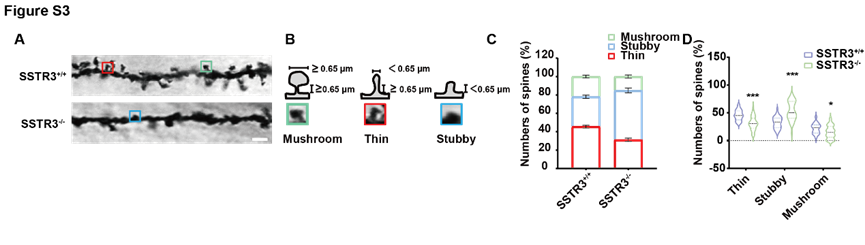


**Figure S3.** **SSTR3 knockout reduced mature dendritic spines.**


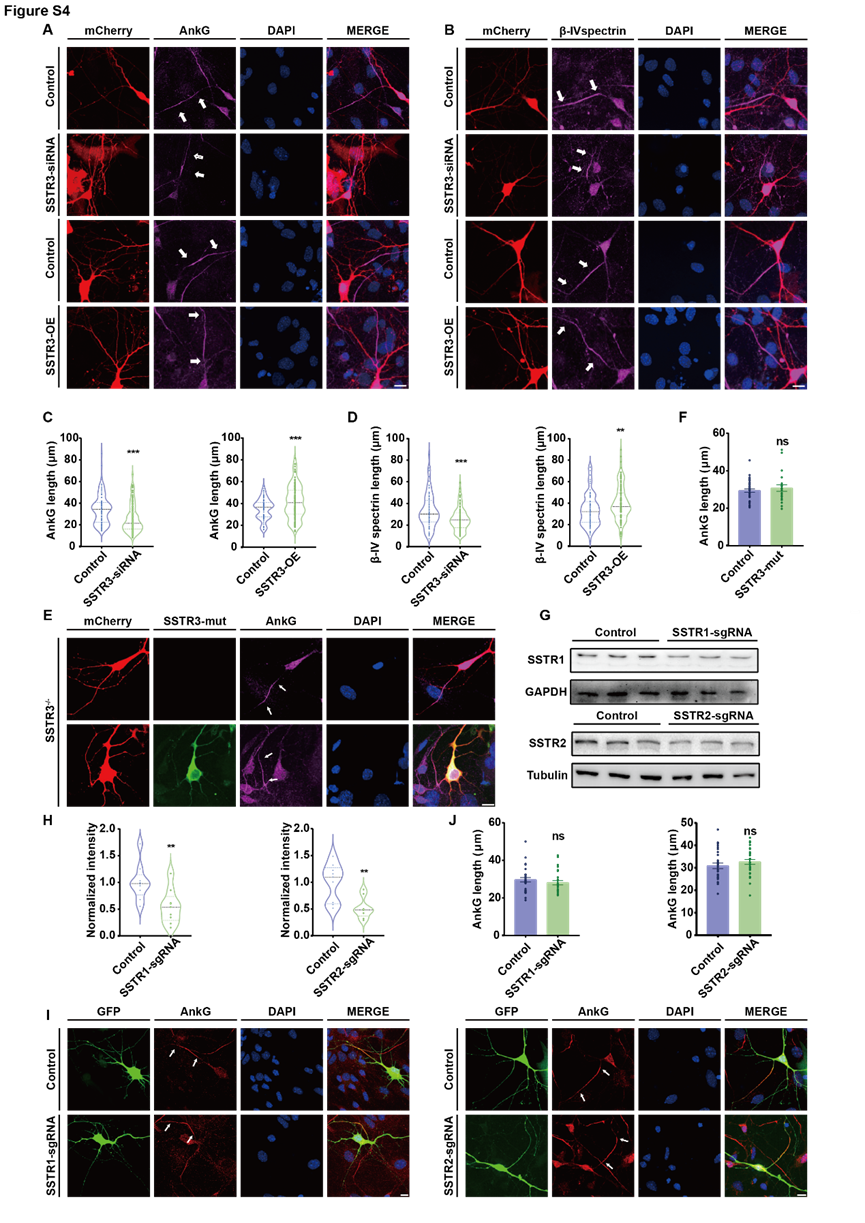


**Figure S4.** **SSTR3 alteration changed AIS length.**


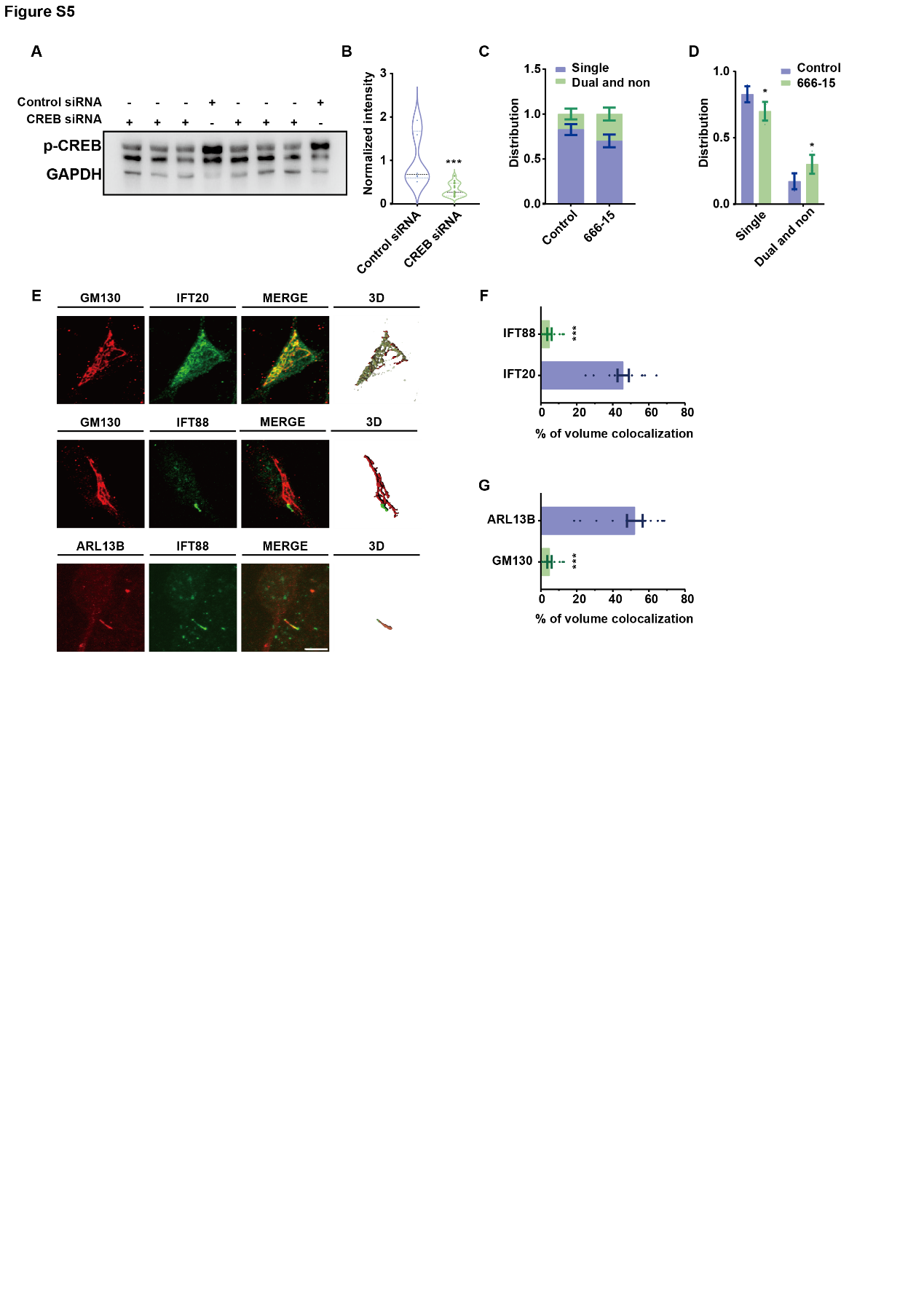


**Figure S5. CREB expression affected AIS structure.**

**SI Figure legends**

**Figure S1.** **Disruption of SSTR3 resulted in changes in cilia length.**

A, Both endogenous and exogenous SSTR3 were localized on the primary cilia in HEK293T cells. Primary ciliary immunofluorescence image of HEK293T. Top, primary cilia were marked using AC3 (red). SSTR3 (green) and AC3 could be co-located, and DAPI (blue) marked the nuclei. Middle, 5-HT6R (red) to labeled the primary cilia. SSTR3 (green) and 5-HT6R (red) were co-located, and DAPI (blue) labeled the nuclei. Bottom, the primary cilia were labeled with 5-HT6R (red), the externally transfected SSTR3-GFP (green) and 5-HT6R (red) could be co-located, and DAPI (blue) labeled the nuclei. Scale bar, 10 μm.

B-C, Function of SSTR3 in regulating ciliary morphology was related to its ciliary localization. Hippocampal neurons were transfected with control, SSTR3-GFP and two point-mutant plasmids (S341A S346A S351A T357A and A243F Q247F A251F Q255F F329A K330A), respectively. B. Primary ciliary immunofluorescence image in neurons transfected with plasmids. mCherry (red) indicated neuron morphology, AC3 (magenta) marked primary cilia, and DAPI (blue) marked the nuclei. Scale bar, 10 μm. C. Statistical data of ciliary length of neurons with control, SSTR3-GFP and two point-mutant plasmids transfection. One-way ANOVA followed by Dunnett post-hoc test was based on data from three independent experiments; F _(3, 358)_ = 157.5; control, n = 97; SSTR3-GFP, n = 83; S341A S346A S351A T357A, n = 89; A243F Q247F A251F Q255F F329A K330A, n = 93.

D-E, The siRNA silencing effects of SSTR3. D. Western blotting of the siRNA knockdown effects of SSTR3 in HEK293T cells using GAPDH as a reference. E. Quantitative analysis of western blotting results. One-way ANOVA followed by Dunnett post-hoc test was based on data from three independent experiments; ****p* < 0.001, **p* = 0.011, F _(4, 40)_ = 6.162. The data were normalized according to control group.

F-G, In the SSTR family, only knocking down SSTR3 expression can shorten cilia length. F. Immunofluorescence images of cilia after transfected with SSTR1-SSTR5 siRNA in mouse hippocampal neurons, respectively. GFP (green) indicated the morphology of neurons, AC3 (red) marked the primary cilia, and DAPI (blue) marked the nuclei. Scale bar, 10 μm. G. Statistical data of ciliary length after transfected with control and SSTR1-5 siRNA. One-way ANOVA followed by Dunnett post-hoc test was based on data from three independent experiments; *p* < 0.001; F _(5, 604)_ = 4.795; control, n = 90; SSTR1-siRNA, n = 101; SSTR2-siRNA, n = 103; SSTR3-siRNA, n = 105; SSTR4-siRNA, n = 108; SSTR5-siRNA, n = 103.

H, There was no SSTR3-positive neurons in the SSTR3^-/-^ mouse brain slices. Immunohistochemical images of SSTR3-positive primary cilia in the hippocampus of SSTR3^+/+^ and SSTR3^-/-^ mice. SSTR3 (red) indicated SSTR3-positive neuron cilia, and DAPI (blue) marked the nucleus. Scale bar, 10 μm. Enlarged view scale bar, 5 μm.

**Figure S2. SSTR3 deletion did not affect the normal expression of SHH signaling pathway.**

A-B, There was no significant difference in the expression level of Gli1, Gli2, Gli3FL and Gli3R in the hippocampus of SSTR3^+/+^ and SSTR3^-/-^ mice. A. Western blotting of Gli1, Gli2, Gli3FL and Gli3R expression in SSTR3^+/+^ and SSTR3^-/-^ mouse brain tissue, using GAPDH as a reference. B. Quantitative analysis of Gli1, Gli2, Gli3FL and Gli3R expression. Left, *p* = 0.692; Middle, *p* = 0.778; Right, *p* = 0.386; n = 9 for each group. Unpaired t test, data from 3 independent experiments. The data were normalized according to SSTR3^+/+^ group.

C-D, There was no significant difference in the expression level of Gli1, Gli2, Gli3FL and Gli3R in the MEF of SSTR3^+/+^ and SSTR3^-/-^ mice. C, Western blotting of Gli1, Gli2, Gli3FL and Gli3R expression in SSTR3^+/+^ and SSTR3^-/-^ MEF, using GAPDH as a reference. D, Quantitative analysis of Gli1, Gli2, Gli3FL and Gli3R expression. Left, *p* = 0.726; Middle, *p* = 0.531; Right, *p* = 0.248; n = 9 for each group. Unpaired t test, data from 3 independent experiments. The data were normalized according to SSTR3^+/+^ group.

**Figure S3.** **SSTR3 knockout reduced mature dendritic spines.**

A, The dendritic spines of Golgi staining in the hippocampus of SSTR3^+/+^ and SSTR3^-/-^ mice. Scale bar: 5 μm.

B, Schematic representation of different dendritic spines.

C-D, Percentage of mature spines remarkably decreased in the hippocampus neurons of SSTR3^-/-^ mice. C. The proportion of differentiated dendritic spines in the hippocampus of SSTR3^+/+^ and SSTR3^-/-^ mice. The results were represented by mean ± SEM. D. Statistical results of hippocampal dendritic spines in mice. The ratio of mushroom and thin spines in SSTR3^-/-^ mice decreased significantly, while the ratio of stubby spines increased significantly. Two-way ANOVA followed by Sidak post-hoc test; F _(2, 246)_ = 49.96; Thin, *p* <0.001; Stubby, *p* <0.001; Mushroom, *p* = 0.032. SSTR3^+/+^, n = 40; SSTR3^-/-^, n = 44.

**Figure S4.** **SSTR3 alteration changed AIS length.**

A-D, Knockdown of SSTR3 in SSTR3^+/+^ neurons shortened AIS length and overexpression of SSTR3 led AIS become longer. Immunocytochemistry results for AnkG (A) and β-Ⅳ spectrin (B) in the neurons of SSTR3^+/+^ with SSTR3-siRNA transfected or SSTR3 overexpression. mCherry (red) indicated neuron morphology, AnkG and β-Ⅳ spectrin (magenta) marked AIS, and DAPI (blue) marked the nuclei. Scale bar, 10 μm. C-D, Decrease of SSTR3 expression caused the shortening of AIS and the neurons with SSTR3 overexpression possessed longer AIS than control neurons. C. Left, *p* < 0.001; control, n = 99; SSTR3-siRNA, n = 105. Right, *p* < 0.0001; control, n = 101; SSTR3-OE, n = 107. D, Left, *p* < 0.001; control, n = 85; SSTR3-siRNA, n = 88. Right, *p* = 0.002; control, n = 107; SSTR3-OE, n = 113. Unpaired t test, data from 3 independent experiments.

E-F, Function of SSTR3 in regulating AIS length was related to its ciliary localization. Hippocampal neurons were transfected with control and SSTR3 point-mutant plasmids (A243F Q247F A251F Q255F F329A K330A), respectively. E. Immunocytochemistry results for AIS in SSTR3^-/-^ neurons with transfections. mCherry (red) indicated neuron morphology, AnkG (magenta) marked AIS, and DAPI (blue) marked the nuclei. Scale bar, 10 μm. F. Statistical data of AIS length of neurons with control, SSTR3 point-mutant plasmids transfection. *p* = 0.447; control, n = 38; SSTR3-mut, n = 23; Unpaired t test, data from 3 independent experiments. The results were represented by mean ± SEM.

G-H, The sgRNA silencing effects of SSTR1 and SSTR2. G. Western blotting of the sgRNA knockdown effects of SSTR1 and SSTR2 in SY5Y cells using GAPDH and β-tubulin as reference. H. Quantitative analysis of western blotting results. Control vs. SSTR1-sgRNA, *p* = 0.010; control vs. SSTR2-sgRNA, *p* = 0.002. Unpaired t test, data from 3 independent experiments. The data were normalized according to control group.

I-J, SSTR1 and SSTR2 knockdown did not regulate AIS length. I. Immunocytochemistry results for AIS in SSTR1-sgRNA or SSTR2-sgRNA transfected neurons. GFP (green) indicated neuron morphology, AnkG (red) marked AIS, and DAPI (blue) marked the nuclei. Scale bar, 10 μm. J. Statistical data of AIS length of neurons with control, SSTR1-sgRNA and SSTR2-sgRNA transfection. Control vs. SSTR1-sgRNA, *p* = 0.350; control, n = 32; SSTR1-sgRNA, n = 27; control vs. SSTR2-sgRNA, *p* = 0.295; control, n = 31; SSTR1-sgRNA, n = 30. Unpaired t test, data from 3 independent experiments. The results were represented by mean ± SEM.

**Figure S5. CREB expression affected AIS structure.**

A-B, The siRNA silencing effects of CREB. A. Western blotting of the siRNA knockdown effects of p-CREB in HT22 cells using GAPDH as a reference. B Quantitative analysis of western blotting results, *p* <0.001. Unpaired t test, data from 3 independent experiments. The data were normalized according to control group.

C-D, 666-15 treatment impaired AIS periodic structure. C. The proportion of different distributions with the treatment of control and 666-15. The results were represented by mean ± SEM. D. Statistical results of different distributions. The ratio of single-peak periodic structure decreased significantly. Two-way ANOVA followed by Sidak post-hoc test was based on data from three independent experiments; F _(1, 12)_ = 15.22. Single, *p* = 0.026. Dual and non, *p* = 0.026. The results were represented by mean ± SEM.

E. Immunofluorescence analysis of IFT20 and IFT88 localization in neurons with antibodies against GM130 or ARL13B. The 3D reconstructions based on sequential 0.2 µm sections were shown on the last panel of each row. Scale bar, 10 µm.

F. Quantification of the volume colocalization (as percentage) of the different IFT proteins with the GM130 staining. *p* < 0.001; IFT88, n = 13; IFT20, n = 13. Unpaired t test, the results were represented by mean ± SEM.

G. Quantification of the volume colocalization (as percentage) of the different subcellular organelles with the IFT88 staining. *p* < 0.001; ARL13B, n = 15; GM130, n = 13. Unpaired t test, the results were represented by mean ± SEM.
